# Bi-allelic *ATG4D* variants are associated with a neurodevelopmental disorder characterized by speech and motor impairment

**DOI:** 10.1038/s41525-022-00343-8

**Published:** 2023-02-10

**Authors:** Marie Morimoto, Vikas Bhambhani, Nour Gazzaz, Mariska Davids, Paalini Sathiyaseelan, Ellen F. Macnamara, Jennifer Lange, Anna Lehman, Patricia M. Zerfas, Jennifer L. Murphy, Maria T. Acosta, Camille Wang, Emily Alderman, Undiagnosed Diseases Network, Margaret Adam, Raquel L. Alvarez, Justin Alvey, Laura Amendola, Ashley Andrews, Euan A. Ashley, Mahshid S. Azamian, Carlos A. Bacino, Guney Bademci, Ashok Balasubramanyam, Dustin Baldridge, Jim Bale, Michael Bamshad, Deborah Barbouth, Pinar Bayrak-Toydemir, Anita Beck, Alan H. Beggs, Edward Behrens, Gill Bejerano, Hugo J. Bellen, Jimmy Bennett, Beverly Berg-Rood, Jonathan A. Bernstein, Gerard T. Berry, Anna Bican, Stephanie Bivona, Elizabeth Blue, John Bohnsack, Devon Bonner, Lorenzo Botto, Brenna Boyd, Lauren C. Briere, Elly Brokamp, Gabrielle Brown, Elizabeth A. Burke, Lindsay C. Burrage, Manish J. Butte, Peter Byers, William E. Byrd, John Carey, Olveen Carrasquillo, Thomas Cassini, Ta Chen Peter Chang, Sirisak Chanprasert, Hsiao-Tuan Chao, Gary D. Clark, Terra R. Coakley, Laurel A. Cobban, Joy D. Cogan, Matthew Coggins, F. Sessions Cole, Heather A. Colley, Cynthia M. Cooper, Heidi Cope, William J. Craigen, Andrew B. Crouse, Michael Cunningham, Precilla D’Souza, Hongzheng Dai, Surendra Dasari, Joie Davis, Jyoti G. Dayal, Esteban C. Dell’Angelica, Katrina Dipple, Daniel Doherty, Naghmeh Dorrani, Argenia L. Doss, Emilie D. Douine, Laura Duncan, Dawn Earl, David J. Eckstein, Lisa T. Emrick, Christine M. Eng, Cecilia Esteves, Marni Falk, Elizabeth L. Fieg, Paul G. Fisher, Brent L. Fogel, Irman Forghani, Ian Glass, Bernadette Gochuico, Page C. Goddard, Rena A. Godfrey, Katie Golden-Grant, Alana Grajewski, Irma Gutierrez, Don Hadley, Sihoun Hahn, Meghan C. Halley, Rizwan Hamid, Kelly Hassey, Nichole Hayes, Frances High, Anne Hing, Fuki M. Hisama, Ingrid A. Holm, Jason Hom, Martha Horike-Pyne, Alden Huang, Sarah Hutchison, Wendy J. Introne, Rosario Isasi, Kosuke Izumi, Fariha Jamal, Gail P. Jarvik, Jeffrey Jarvik, Suman Jayadev, Orpa Jean-Marie, Vaidehi Jobanputra, Lefkothea Karaviti, Jennifer Kennedy, Shamika Ketkar, Dana Kiley, Gonench Kilich, Shilpa N. Kobren, Isaac S. Kohane, Jennefer N. Kohler, Susan Korrick, Mary Kozuira, Deborah Krakow, Donna M. Krasnewich, Elijah Kravets, Seema R. Lalani, Byron Lam, Christina Lam, Brendan C. Lanpher, Ian R. Lanza, Kimberly LeBlanc, Brendan H. Lee, Roy Levitt, Richard A. Lewis, Pengfei Liu, Xue Zhong Liu, Nicola Longo, Sandra K. Loo, Joseph Loscalzo, Richard L. Maas, Calum A. MacRae, Valerie V. Maduro, Rachel Mahoney, Bryan C. Mak, Laura A. Mamounas, Teri A. Manolio, Rong Mao, Kenneth Maravilla, Ronit Marom, Gabor Marth, Beth A. Martin, Martin G. Martin, Julian A. Martínez-Agosto, Shruti Marwaha, Jacob McCauley, Allyn McConkie-Rosell, Alexa T. McCray, Elisabeth McGee, Heather Mefford, J. Lawrence Merritt, Matthew Might, Ghayda Mirzaa, Eva Morava, Paolo Moretti, Mariko Nakano-Okuno, Stanley F. Nelson, John H. Newman, Sarah K. Nicholas, Deborah Nickerson, Shirley Nieves-Rodriguez, Donna Novacic, Devin Oglesbee, James P. Orengo, Laura Pace, Stephen Pak, J. Carl Pallais, Christina G. S. Palmer, Jeanette C. Papp, Neil H. Parker, John A. Phillips, Jennifer E. Posey, Lorraine Potocki, Barbara N. Pusey Swerdzewski, Aaron Quinlan, Deepak A. Rao, Anna Raper, Wendy Raskind, Genecee Renteria, Chloe M. Reuter, Lynette Rives, Amy K. Robertson, Lance H. Rodan, Jill A. Rosenfeld, Natalie Rosenwasser, Francis Rossignol, Maura Ruzhnikov, Ralph Sacco, Jacinda B. Sampson, Mario Saporta, Judy Schaechter, Timothy Schedl, Kelly Schoch, Daryl A. Scott, C. Ron Scott, Vandana Shashi, Jimann Shin, Edwin K. Silverman, Janet S. Sinsheimer, Kathy Sisco, Edward C. Smith, Kevin S. Smith, Emily Solem, Lilianna Solnica-Krezel, Benjamin Solomon, Rebecca C. Spillmann, Joan M. Stoler, Kathleen Sullivan, Jennifer A. Sullivan, Angela Sun, Shirley Sutton, David A. Sweetser, Virginia Sybert, Holly K. Tabor, Queenie K.-G. Tan, Amelia L. M. Tan, Mustafa Tekin, Fred Telischi, Willa Thorson, Camilo Toro, Alyssa A. Tran, Rachel A. Ungar, Tiina K. Urv, Adeline Vanderver, Matt Velinder, Dave Viskochil, Tiphanie P. Vogel, Colleen E. Wahl, Melissa Walker, Stephanie Wallace, Nicole M. Walley, Jennifer Wambach, Jijun Wan, Lee-Kai Wang, Michael F. Wangler, Patricia A. Ward, Daniel Wegner, Monika Weisz Hubshman, Mark Wener, Tara Wenger, Katherine Wesseling Perry, Monte Westerfield, Matthew T. Wheeler, Jordan Whitlock, Lynne A. Wolfe, Kim Worley, Changrui Xiao, Shinya Yamamoto, John Yang, Zhe Zhang, Stephan Zuchner, Sara Reichert, Audrey Thurm, David R. Adams, Wendy J. Introne, Sharon M. Gorski, Cornelius F. Boerkoel, William A. Gahl, Cynthia J. Tifft, May Christine V. Malicdan

**Affiliations:** 1 National Institutes of Health Undiagnosed Diseases Program, Common Fund, Office of the Director, National Institutes of Health, Bethesda, MD 20892 USA; 2 Department of Medical Genetics, Children’s Hospitals and Clinics of Minnesota, Minneapolis, MN 55404 USA; 3 Department of Medical Genetics, Faculty of Medicine, University of British Columbia, Vancouver, BC V6H 3N1 Canada; 4 Provincial Medical Genetics Program, British Columbia Women’s and Children’s Hospital, Vancouver, BC V6H 3N1 Canada; 5 Department of Pediatrics, Faculty of Medicine, King Abdulaziz University, Jeddah, Saudi Arabia; 6 Canada’s Michael Smith Genome Sciences Centre, BC Cancer, Vancouver, BC V5Z 1L3 Canada; 7 Department of Molecular Biology and Biochemistry, Simon Fraser University, Burnaby, BC V5A 1S6 Canada; 8 Diagnostic and Research Services Branch, Office of Research Services, National Institutes of Health, Bethesda, MD 20892 USA; 9 Neurodevelopmental and Behavioral Phenotyping Service, Office of the Clinical Director, National Institute of Mental Health, National Institutes of Health, Bethesda, MD 20892 USA; 10 Office of the Clinical Director, National Human Genome Research Institute, National Institutes of Health, Bethesda, MD 20892 USA; 11 Human Biochemical Genetics Section, Medical Genetics Branch, National Human Genome Research Institute, National Institutes of Health, Bethesda, MD 20892 USA; 12 Pacific Northwest Undiagnosed Diseases Clinical Site at University of Washington and Seattle Children’s Hospital, Seattle, WA USA; 13 Center for Undiagnosed Diseases at Stanford, Stanford, CA USA; 14 University of Utah, Salt Lake City, UT USA; 15 Baylor College of Medicine, Houston, TX USA; 16 University of Miami School of Medicine, Miami, FL USA; 17 Washington University in St. Louis, St. Louis, MO USA; 18 Associated Regional and University Pathologists Laboratories, Salt Lake City, UT 84108 USA; 19 UDN Clinical Site at Harvard Medical School, Boston, MA USA; 20 Children’s Hospital of Philadelphia, Philadelphia, PA USA; 21 Vanderbilt Center for Undiagnosed Diseases, Nashville, TN USA; 22 UCLA Undiagnosed Diseases Clinic, Los Angeles, CA USA; 23 University of Alabama at Birmingham, Birmingham, AL USA; 24 Duke University, Durham, NC USA; 25 Mayo Clinic, Rochester, MN USA; 26 Harvard Medical School, Boston, MA USA; 27 Columbia University Irving Medical Center, New York, NY USA; 28 University of Pennsylvania, Philadelphia, PA USA; 29 University of Oregon, Eugene, OR USA

**Keywords:** Medical genomics, Disease genetics

## Abstract

Autophagy regulates the degradation of damaged organelles and protein aggregates, and is critical for neuronal development, homeostasis, and maintenance, yet few neurodevelopmental disorders have been associated with pathogenic variants in genes encoding autophagy-related proteins. We report three individuals from two unrelated families with a neurodevelopmental disorder characterized by speech and motor impairment, and similar facial characteristics. Rare, conserved, bi-allelic variants were identified in *ATG4D*, encoding one of four ATG4 cysteine proteases important for autophagosome biogenesis, a hallmark of autophagy. Autophagosome biogenesis and induction of autophagy were intact in cells from affected individuals. However, studies evaluating the predominant substrate of ATG4D, GABARAPL1, demonstrated that three of the four ATG4D patient variants functionally impair ATG4D activity. GABARAPL1 is cleaved or “primed” by ATG4D and an in vitro GABARAPL1 priming assay revealed decreased priming activity for three of the four ATG4D variants. Furthermore, a rescue experiment performed in an *ATG4* tetra knockout cell line, in which all four ATG4 isoforms were knocked out by gene editing, showed decreased GABARAPL1 priming activity for the two ATG4D missense variants located in the cysteine protease domain required for priming, suggesting that these variants impair the function of ATG4D. The clinical, bioinformatic, and functional data suggest that bi-allelic loss-of-function variants in *ATG4D* contribute to the pathogenesis of this syndromic neurodevelopmental disorder.

## Introduction

Autophagy is a dynamic and highly conserved process that regulates the degradation and recycling of cellular components. Three distinct types of autophagy include macroautophagy, microautophagy, and chaperone-mediated autophagy^1^. Macroautophagy (hereafter referred to as autophagy) involves the formation of a double-membraned organelle called the autophagosome that delivers cytoplasmic components to lysosomes for degradation, while microautophagy and chaperone-mediated autophagy are autophagosome-independent pathways by which cytoplasmic components undergo direct engulfment by the lysosome or are selectively diverted to the lysosome by chaperones, respectively^1,2^. Upregulation of autophagy occurs when cells require nutrients and energy during starvation, during cell state or developmental transitions, or when cells need to degrade cytoplasmic components such as damaged cellular macromolecules or organelles, pathogens, and protein aggregates accumulated through processes such as oxidative stress, infection, disease, and aging^1,3^. Defective autophagy has been shown to contribute to the pathogenesis of various human diseases, including rare monogenic disorders and common complex diseases such as neurodegenerative diseases, heart diseases, and cancer^1,3^.

Autophagosome biogenesis requires the coordinated action of approximately 20 core AuTophaGy-related (ATG) proteins^4,5^. These core ATG proteins regulate the key steps of autophagosome biogenesis, including initiation, membrane nucleation, membrane expansion, and closure; this process is considered to be a marker of autophagy^3,4^. Integration of Atg8 into the pre-autophagosomal structure (in yeast) or phagophore (in mammals) is important for membrane expansion, cargo sequestration, and autophagosome-lysosome fusion^4,6^. The mammalian orthologs of yeast Atg8 include six proteins from two subfamilies: the microtubule-associated proteins 1A/1B light chain 3 (LC3) subfamily (LC3A, LC3B, and LC3C) and the GABA type A receptor-associated protein (GABARAP) subfamily (GABARAP, GABARAPL1, and GABARAPL2)^4^; these proteins have both shared and unique roles in membrane expansion, cargo specificity, and lysosomal fusion^6–10^.

The ATG4 family of cysteine proteases is thought to be involved in processing the LC3/GABARAP subfamilies. Specifically, the ATG4 cysteine proteases prime the LC3/GABARAP subfamily proteins by cleaving pro-LC3/GABARAP into LC3/GABARAP-I to expose a C-terminal glycine residue that can then be lipidated by conjugation to phosphatidylethanolamine (PE) in the phagophore membrane to form LC3/GABARAP-II^11^. This lipidated form can then bind p62 and other receptor proteins for selective autophagy^12^. The ATG4 cysteine proteases also delipidate the LC3/GABARAP subfamily proteins^13^. While there is a single Atg4 in yeast, there are four homologs of ATG4 in mammals, i.e., ATG4A, ATG4B, ATG4C, and ATG4D that are functionally redundant^14^. These homologs have differences in priming activity and substrate specificity: ATG4B has the highest priming activity and broadest specificity with the ability to prime all LC3/GABARAP subfamily proteins; ATG4A has a lower priming activity compared to ATG4B with specificity toward the GABARAP subfamily; and ATG4C and ATG4D have weak priming activity although cleavage of the N-terminal domain of ATG4D increases its priming activity for GABARAPL1^15,16^. In contrast, delipidation of the LC3/GABARAP subfamily proteins is mediated by all four ATG4 protein isoforms in in vitro studies^13,17^. Additionally, the ATG4 proteins stabilize LC3/GABARAP proteins and promote phagophore growth independent of their protease activity^18,19^. Interestingly, several autophagy-independent functions for the ATG4 family have also emerged, including roles in protein stabilization^18,19^, receptor trafficking^20^, mitochondrial biology^13,15,21^, and phosphatidylserine delipidation in non-canonical autophagy^17^.

Several model organisms deficient for the ATG4 protein isoforms have been associated with neuropathology and emphasize a role for these proteins in the central nervous system. In *Atg4b*-deficient mice, mild motor performance deficits and histological findings of spheroid-like bodies containing amorphous proteinaceous aggregates in the deep cerebellar and vestibular nuclei were observed^22^. In *Atg4d*-deficient mice, age-dependent cerebellar neurodegeneration and motor dysfunction were observed^20^. In a dog model with a homozygous missense variant in *Atg4d*, progressive cerebellar ataxia and a corresponding loss of cerebellar Purkinje cells were seen^23^. Further, in a zebrafish *atg4da* knockdown model, loss of cerebellar neurons was also observed^23^. These findings suggest that the ATG4 family of cysteine proteases are required for the proper maintenance and function of the cerebellum.

The development of next-generation sequencing, the application of tools for collaboration such as GeneMatcher^24^, and the implementation of large-scale collaborative efforts such as the Undiagnosed Diseases Network (UDN)^25–28^ and other similar clinical and collaborative efforts have facilitated the discovery of Mendelian disorders and improved the diagnoses and care of individuals with ultra-rare disorders and conditions. While genetic variants in the genes encoding the ATG4 protein isoforms have been linked to cancer and inflammation^29^, monogenic disorders have not been associated with any of the genes encoding the ATG4 family of cysteine proteases so far. In this study, we report three individuals from two unrelated families presenting with a neurodevelopmental disorder associated with bi-allelic variants in *ATG4D*.

## Results

### Identification of a neurodevelopmental disorder characterized by speech and motor impairment

**Fig. 1:**
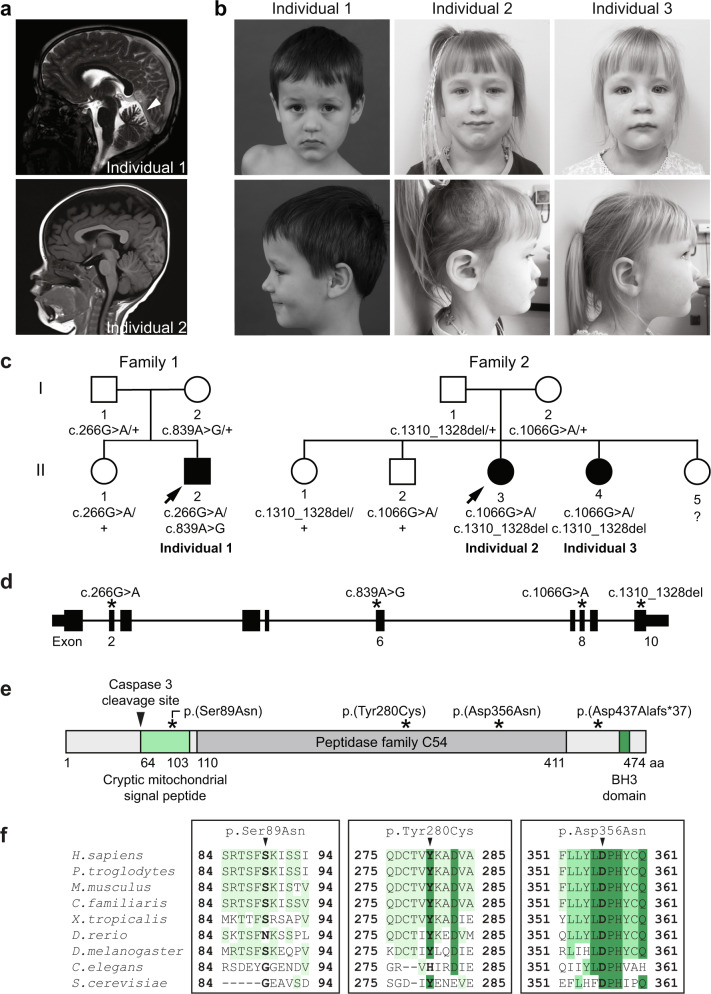
Bi-allelic variants in *ATG4D* segregate with a neurodevelopmental disorder in three individuals from two unrelated families. **a** Neuroimaging of Individual 1 showing mild cerebellar atrophy disproportionately involving the superior cerebellar hemispheres and vermis at 5 years 3 months (arrowhead in T2-weighted sagittal view image, left panel). There were no remarkable neuroimaging findings for Individual 2 at 1 year 3 months (arrow in T1-weighted sagittal view image, right panel). **b** Photographs of the affected individuals showing a common facial gestalt characterized by almond-shaped eyes, a depressed nasal bridge, and a prominent Cupid’s bow. Age at the time of the photos is 5 years 3 months for Individual 1, 4 years 8 months for Individual 2, and 3 years 5 months for Individual 3. Written consent was obtained for the publication of photographs. **c** The pedigrees of two unrelated families with at least one affected individual exhibiting a neurodevelopmental disorder show segregation of compound heterozygous *ATG4D* variants with disease. **d** Schematic of the *ATG4D* gene showing the relative location of each variant (asterisks). The schematic is to scale, while the variant position is approximate. **e** Schematic of the ATG4D cysteine protease and its functional domains including the peptidase family C54 domain (dark gray), a cryptic mitochondrial signal peptide (light green), a BH3 domain (green), and a caspase 3 cleavage site (arrowhead). The location of the predicted amino acid changes is indicated (asterisks). The schematic and variant position are to scale. **f** Alignment of missense variants in ATG4D across multiple species including human (*Homo sapiens*), chimpanzee (*Pan troglodytes*), mouse (*Mus musculus*), dog (*Canine familiaris*), African clawed frog (*Xenopus laevis*), zebrafish (*Danio rerio*), fruit fly (*Drosophila melanogaster*), nematode (*Caenhabdoritis elegans*), and budding yeast (*Saccharomyces cerevisiae*). The amino acid residue of interest is indicated by the arrowhead and the level of conservation is indicated by the intensity of the color.

Individual 1 (Family 1: II-2) first presented for evaluation at 3 years 4 months due to an abnormal gait, poor coordination, and staring episodes. He is the second child of healthy non-consanguineous parents. He has an unaffected sister who had received a preliminary diagnosis of pervasive developmental disorder which has been resolved; she is currently 15 years of age and doing well academically. The family history was otherwise unremarkable. His early development was considered within normal limits, and he began independently walking at 13 months. At 2 years, generalized low tone, frequent tripping and falling, and frequent staring episodes were observed. At 3 years 2 months, his mother described recurrent events characterized by ataxia, dysarthria, confusion, and behavioral changes that lasted approximately 20 min. A neurological evaluation revealed hypotonia, decreased deep tendon reflexes, distal muscle weakness, a wide-based and uncoordinated gait, and reduced verbal interaction (60-75% intelligible). At 3 years 6 months, he had an absence seizure and an EEG revealed multifocal abnormalities with high risk for generalized and focal seizures. At 4 years 6 months, he continued to have occasional seizures despite treatment with AED. At 5 years, saccadic intrusions and central nystagmus, dysarthria, intention tremor, poor fine motor coordination, and visual-spatial difficulties were noted. At 9.5 years, mild cognitive impairment, ADHD, and oppositional defiant disorder were noted. Brain magnetic resonance imaging (MRI) at the ages of 3, 3.5, 4.5, 5, and 8 years revealed mild cerebellar atrophy of the superior cerebellar hemispheres and vermis that has remained stable (Fig. 1a).

Individual 2 (Family 2: II-3) presented for evaluation due to abnormal hand movements and staring episodes starting at age 9 months. She is the third child of healthy non-consanguineous Russian parents, and she has three unaffected siblings (an older sister with repaired isolated cleft palate, an older brother, and a younger sister) and an affected sibling (Individual 3 (Family 2: II-4)). She had early gross motor developmental delay, crawling at 12 months and independently walking at 20 months. A brain MRI at 1 year 3 months showed no remarkable findings (Fig. 1a). At 2 years of age, she presented with an abnormal shuffling gait with frequent tripping and falling. Deep tendon reflexes were reduced but present in the patella. She had speech delay with only 3 poorly articulated words. At 2 years 10 months, she was making steady developmental gains, but remained globally delayed. She could run for short distances but tripped easily. Neurological examination normalized by 4 years 8 months, her abnormal hand movements and gait resolved, and her gross motor skills were considered within normal limits. She has a mild learning disability and poor speech articulation.

Individual 3 (Family 2: II-4), the younger sister of Individual 2, was born at 32 weeks and 5 days of gestation via Cesarean section due to vaginal bleeding, preterm labor, and fetal distress. She was admitted to the NICU for 4 weeks for management of prematurity, transient respiratory distress, and jaundice. She had mild speech and gross motor delay, which improved and normalized by 3 years 5 months, though speech articulation remains poor.

A summary of the clinical findings is presented in Table 1 and a detailed clinical history is available in the Supplementary Information. Of note, all three affected individuals presented with a similar facial gestalt comprising almond-shaped eyes, depressed nasal bridge, and a prominent Cupid’s bow (Fig. 1b).

**Table 1:** Summary of clinical features of individuals with bi-allelic *ATG4D* variants.

Clinical feature	Individual 1 (Family 1: II-2)	Individual 2 (Family 2: II-3)	Individual 3 (Family 2: II-4)
*Demographics*			
Sex	Male	Female	Female
Ethnicity	European	Tatar/Russian	Tatar/Russian
Age of onset	2 years	9 months	18 months
Chief complaint	Episodic dyscoordination and gait abnormality, staring episodes, and mild speech and motor developmental delay	Abnormal hand movements and staring episodes (concern for seizures), gait abnormality, mild speech and motor developmental delay	Mild speech and motor developmental delay
*Prenatal and perinatal history*			
Gestation at delivery	Full term (40 weeks)	Early term (37 weeks)	Preterm (32 weeks and 5 days)
Delivery	Vaginal delivery (pitocin induction)	Elective C-section	C-section for preterm labor and fetal distress
Birth weight	3500 g (44th percentile)	3500 g (91st percentile)	2263 g (86th percentile)
Birth length	53.0 cm (79th percentile)	NA	46.0 cm (92nd percentile)
Head circumference	36.2 cm (79th percentile)	NA	32.5 cm (98th percentile)
*Growth parameters*			
Age at measurement	5 years 3 months	4 years 8 months	3 years 5 months
Weight	20.7 kg (74th percentile)	16 kg (29th percentile)	15 kg (58th percentile)
Height	114.0 cm (77th percentile)	107.6 cm (69th percentile)	96.0 cm (42nd percentile)
*Physical findings*			
Characteristic facial features (almond-shaped eyes, depressed nasal bridge, and prominent Cupid’s bow)	+	+	+
Auricular tag	+ (left)	−	−
*Ophthalmological findings*			
Hyperopia, amblyopia, astigmatism	+	−	−
*Musculoskeletal findings*			
Muscular hypotonia	+	WNL	WNL
Distal muscle weakness	+ (progressive, age at onset: 3 years)	Not observed^a^	Not observed^a^
*Neurological findings*			
Abnormal gross motor development	+ (slow, progressive decline)	+/− (delayed: crawled at 12 months, walked at 20 months, ran a little at 2 years 10 months; resolved by 4 years 8 months)	+ (delayed; walked at 19 months)
Speech impairment	+ (speech delay and dysarthria)	+ (speech delay with 1st word at 12 months/3 words at 21 months; poor speech articulation at 4 years 8 months)	+ (speech delay with 1st word at 12 months/2 words at 20 months; poor speech articulation at 3 years 5 months)
Mild cognitive impairment	+	+	Not observed^a^
Abnormal gait	+ (frequent falls and clumsiness, age at onset: 2 years; progressive unsteady gait, age at onset: 4 years)	+/− (shuffling gait, frequent falls/tripping, and clumsiness at 25 months and at 2 years 10 months, resolved by 4 years 8 months)	Not observed^a^
Abnormal eye movements	+ (saccadic intrusions and central nystagmus)	Not observed^a^	Not observed^a^
Intention tremor	+ (bilateral, progressive)	Not observed^a^	Not observed^a^
Poor fine motor coordination	+	Not observed^a^	Not observed^a^
Decreased DTRs	+ (0 at patella and ankles)	+/− (1+ at patella at 25 months, 2+ at 4 years 8 months)	Not observed^a^
Mild sensory neuropathy	+	Not examined	Not examined
Oppositional defiant disorder	+	Not observed^a^	Not observed^a^
ADHD-PI	+ (diagnosed at 7 years)	Not observed^a^	Not observed^a^
Hypersomnolence	+ (age at onset: 4 years)	Not observed^a^	Not observed^a^
Seizures	+ (initially generalized, followed by focal) (described as focal absence)	+/− (concern due to staring episodes at 25 months, resolved by 4 years 8 months)	Not observed^a^
Abnormal EEG findings	+ (multifocal abnormalities, high risk for focal and generalized epilepsy at 3.5 years; prolonged EEG normal at 5 years 3 months)	WNL at 1 year 8 months	ND
*Neuroimaging findings*			
MRI	Non-progressive mild cerebellar atrophy of the superior cerebellar hemispheres and vermis	WNL at 1 year 3 months	ND
MRS	Elevated choline in 5 of 6 locations	ND	ND

+, present; −, absent; *ADHD-PI* attention deficit hyperactivity disorder, predominantly inattentive presentation; *APGAR* appearance pulse grimace activity respiration, *C-section* Caesarean section, *DTR* deep tendon reflex, *EEG* electroencephalogram, *MRI* magnetic resonance imaging, *MRS* magnetic resonance spectroscopy, *NA* not available, *ND* not done, *WNL* within normal limits.

^a^Not observed at the time of examination.

### Exome and genome sequencing identify bi-allelic variants in *ATG4D*

To identify pathogenic variants underlying their neurodevelopmental disorder, trio exome and quartet genome sequencing was performed on Individual 1 and his parents (and his unaffected sibling for the quartet genome sequencing) and trio exome sequencing was performed on Individual 2 and her parents. Compound heterozygous variants in *ATG4D* (NM_032885.5) were independently identified in the two probands segregating with an autosomal recessive mode of inheritance (Fig. 1c, d, Supplementary Table 1, and Supplementary Fig. 1); Individual 3 was found to have the same compound heterozygous variants as Individual 2 by Sanger sequencing. The *ATG4D* variants identified include three substitutions (NM_032885.5: c.266G>A, NM_032885.5: c.839A>G, and NM_032885.5: c.1066G>A) and a deletion (NM_032885.5: c.1310_1328del) and were either predicted to lead to missense variants in functional domains of the protein or a frameshift variant (Fig. 1e and Supplementary Table 1). The Genome Aggregation Database (gnomAD) allele frequencies of the identified missense *ATG4D* variants ranged from 0.00040% to 0.061% with no homozygotes recorded; CADD phred scores were consistent with deleterious sequence variation (Supplementary Table 1). Furthermore, these variants affect conserved amino acid residues (Fig. 1f) and are predicted to be damaging by multiple bioinformatic algorithms (Supplementary Table 2)^30^. Additional candidate gene variants were detected in Individual 1 (Supplementary Table 3) but were deprioritized (see Supplementary Discussion).

### *ATG4D* mRNA expression and ATG4D protein levels in cultured cells from individuals with bi-allelic *ATG4D* variants are comparable to controls

The *ATG4D* variants identified in the affected individuals are predicted to lead to missense variants or a frameshift variant at the C-terminus of ATG4D and were not expected to decrease *ATG4D* mRNA expression or ATG4D protein levels. To test this hypothesis, we quantified *ATG4D* mRNA expression and ATG4D protein levels in cultured cells from the affected individuals. Relative *ATG4D* mRNA expression in the primary fibroblasts and lymphoblastoid cell lines of affected individuals was comparable to controls (Supplementary Fig. 2A–C). ATG4D protein levels in the primary fibroblasts of affected individuals were comparable to controls (Supplementary Fig. 2d, f); ATG4D protein was undetectable in the lymphoblastoid cell lines (Supplementary Fig. 2E). These findings suggest that the *ATG4D* variants do not affect mRNA expression or protein levels but may have other effects on protein functionality.

### Assessment of autophagy in cultured cells from individuals with bi-allelic *ATG4D* variants

**Fig. 2:**
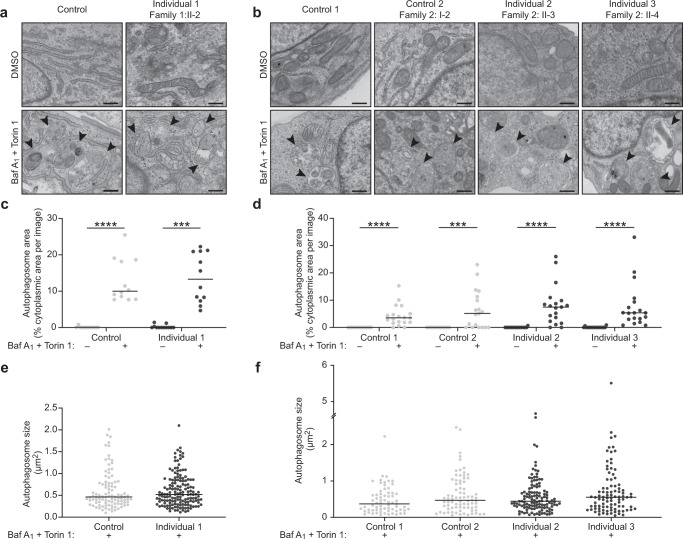
Transmission electron microscopy analyses of autophagosome biogenesis in cultured cells from individuals with bi-allelic variants in *ATG4D*. **a**, **b** Representative TEM images of cultured primary fibroblasts from Control (GM09503) and Individual 1 (**a**) or lymphoblastoid cell lines from Control 1 (CCL-104), Control 2 (Family 2: I-2, mother of Individuals 2 and 3), Individual 2 (Family 2: II-3), and Individual 3 (Family 2: II-4) (B) treated with vehicle (DMSO) or 100 nM Torin 1 and 100 nM Bafilomycin A_1_ for 3 h to induce the formation of autophagosomes and to prevent their degradation, respectively (arrowheads). **c**–**f** Quantification of autophagosome area (**c**, **d**) and size (**e**, **f**) from the experiments represented in (**a**, **b**). The data are presented as dot plots with the median indicated by a horizontal line. Light gray data points represent measurements made on control cells; dark gray data points represent measurements made on affected cells. For the quantification of autophagosome area, 12 images (primary fibroblasts) or 19 images (lymphoblastoid cell lines) taken from each sample were assessed per condition. A Kruskal–Wallis test and Dunn’s multiple comparisons test were performed for relevant predefined dataset pairs. For the quantification of autophagosome size, data points represent individual autophagosomes measured from Torin 1- and Bafilomycin A_1_-treated conditions. For the primary fibroblasts, *n* = 98 and *n* = 152 autophagic structures were respectively measured for Control (GM09503) and Individual 1 (Family 1: II-2), and a two-tailed Mann–Whitney *U* test was performed. For the lymphoblastoid cell lines, *n* = 67, *n* = 78, *n* = 122, and *n* = 92 autophagomsomes were respectively measured for Control 1 (CCL-104), Control 2 (Family 2: I-2, mother of Individuals 2 and 3), Individual 2 (Family 2: II-3), and Individual 3 (Family 2: II-4), and a Kruskal–Wallis test and Dunn’s multiple comparisons test were performed for relevant predefined dataset pairs. Only statistically significant comparisons are shown. Magnification: ×4000. Scale bar: 500 nm. ****p < 0.0001; ***p < 0.001; Baf A_1_, Bafilomycin A_1_; DMSO, dimethyl sulfoxide; TEM, transmission electron microscopy.

Investigation of the specific contribution of each of the four ATG4 cysteine proteases to autophagy has been challenging due to their functional redundancy^13,14,18^. Indeed, gene editing alone or in combination with knockdown strategies of all four ATG4s has been required to further elucidate the common and unique contributions of each ATG4 cysteine protease^13,14,18^, and most studies in genetic animal models have been performed using affected tissues, including neural tissues, from mutant animals^20,23^. Since previous studies have demonstrated that deficiency of one or more of the ATG4 cysteine proteases leads to impaired autophagosome biogenesis^14,18^, we assessed autophagosome biogenesis in cultured cells from the affected individuals by transmission electron microscopy (TEM). The distinct ultrastructure of the autophagosome, a double-membraned compartment encapsulating cytoplasmic material, allows for their identification and quantification^31^. Autophagosome biogenesis was induced in cells with Torin 1 and the autophagy inhibitor Bafilomycin A_1_ was used to prevent their lysosomal degradation, and autophagosome area and size were quantified (Fig. 2). In the vehicle-only treated condition, very few autophagosomes were observed in all samples as expected. In the Torin 1-and Bafilomycin A_1_-treated condition, autophagosomes were comparably induced in the cells from affected individuals and controls. For the primary fibroblasts, the median autophagosome area (% cytoplasmic area per image) in Control was 10.0%, while that of Individual 1 was 13.3% (*p* > 0.05; Fig. 2c); the median autophagosome size in Control was 0.4617 μm^2^, while that of Individual 1 was 0.5177 μm^2^ (*p* > 0.05; Fig. 2e). For the lymphoblastoid cell lines, the median autophagosome areas in Control 1, Control 2 (mother of Individuals 2 and 3), Individual 2, and Individual 3 were 3.55%, 5.15%, 7.48%, and 5.36%, respectively (*p* > 0.05; Fig. 2d); the median autophagosome size in Control 1, Control 2 (mother of Individuals 2 and 3), Individual 2, and Individual 3 were 0.3707 μm^2^, 0.4671 μm^2^, 0.4426 μm^2^, and 0.5519 μm^2^, respectively (*p* > 0.05; Fig. 2f). These data suggest that autophagosome biogenesis is intact in primary fibroblasts and lymphoblastoid cell lines from individuals with bi-allelic variants in *ATG4D*.

**Fig. 3:**
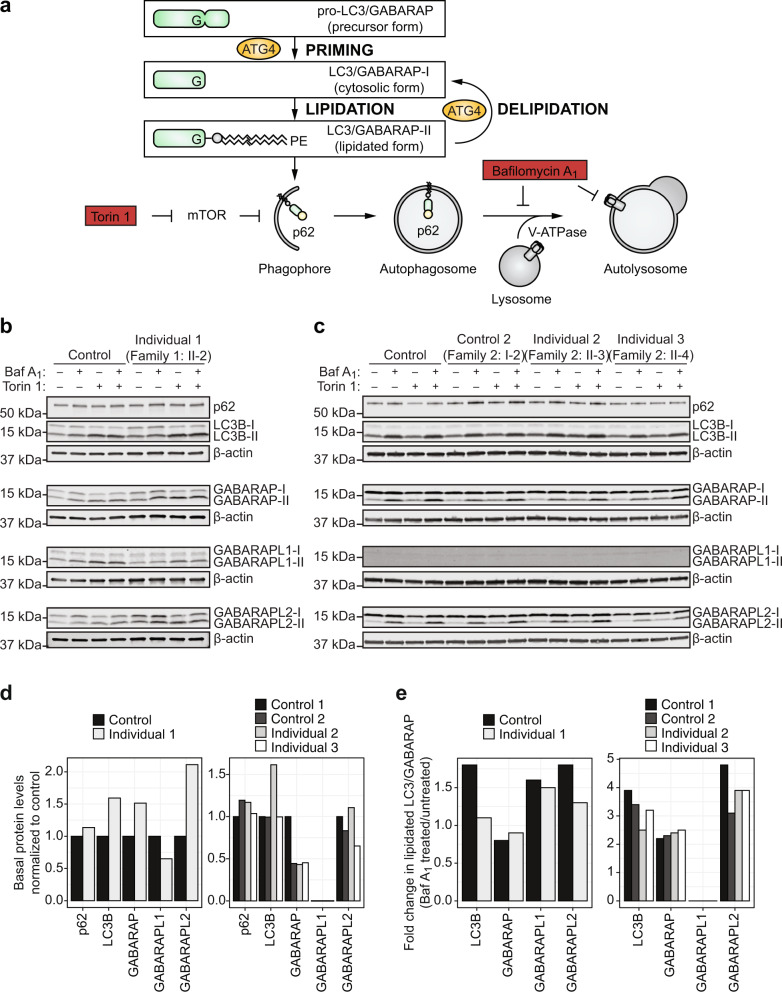
Analysis of the induction of lipidated LC3/GABARAP subfamily proteins in cells from individuals with bi-allelic variants in *ATG4D*. **a** ATG4 family members prime the LC3/GABARAP subfamily proteins required for autophagosome biogenesis by cleaving pro-LC3/GABARAP (precursor form) into LC3/GABARAP-I (cytosolic form) to expose a C-terminal glycine residue that can then be lipidated by conjugation to phosphatidylethanolamine (PE), resident in phagophore membranes, to form LC3/GABARAP-II (lipidated form). This lipidated form in the expanding phagophore membrane can bind p62 and other proteins for selective autophagy. ATG4 family members also cleave LC3/GABARAP-PE to delipidate and recycle LC3/GABARAP to its cytosolic form. Torin 1 is an autophagy inducer and Bafilomycin A_1_ is an autophagy inhibitor. **b** Immunoblot analysis of primary fibroblasts from Control (GM09503) and Individual 1 (Family 1: II-2) assessing p62, LC3B, GABARAP, GABARAPL1, and GABARAPL2 upon induction and/or inhibition of autophagy by treatment with 100 nM Torin 1 and/or 100 nM Bafilomycin A_1_ for 3 h. **c** Immunoblot analysis of lymphoblastoid cell lines from Control 1 (CCL-104), Control 2 (Family 2: I-2, mother of Individuals 2 and 3), Individual 2 (Family 2: II-3), and Individual 3 (Family 2: II-4) assessing p62, LC3B, GABARAP, GABARAPL1, and GABARAPL2 upon induction and/or inhibition of autophagy by treatment with 100 nM Torin 1 and/or 100 nM Bafilomycin A_1_ for 3 h. β-actin was used as a loading control. p62 and LC3B were assessed on the same gel. **d**, **e** Quantification of basal protein levels (**d**) and autophagic flux (**e**) from immunoblot analyses of p62, total LC3B, total GABARAP, total GABARAPL1, and total GABARAPL2 in primary fibroblasts from Control (GM09503) and Individual 1 (Family 1: II-2) and lymphoblastoid cell lines from Control 1 (CCL-104), Control 2 (Family 2: I-2, mother of Individuals 2 and 3), Individual 2 (Family 2: II-3), and Individual 3 (Family 2: II-4). Autophagic flux is presented as the fold change of the lipidated form of each LC3/GABARAP subfamily member after treatment with Bafilomycin A_1_ compared to before treatment. Baf A_1_, Bafilomycin A_1_; kDa, kilodaltons; mTOR, mammalian target of rapamycin.

We next evaluated the autophagy induction and autophagic flux by assessing levels of autophagy markers p62, LC3B-II, GABARAP-II, GABARAPL1-II, and GABARAPL2-II in cells treated with Torin 1 and/or Bafilomycin A_1_. ATG4 family members prime the LC3/GABARAP subfamily proteins by cleaving pro-LC3/GABARAP into LC3/GABARAP-I to expose a C-terminal glycine residue that can then be lipidated by conjugation to phosphatidylethanolamine in the phagophore membrane to form LC3/GABARAP-II (Fig. 3a). The lipidated membrane-bound form can be distinguished from the non-lipidated cytosolic form by SDS-PAGE since the lipid functional group leads to increased hydrophobicity and faster migration^32^. Basal levels of p62 and total LC3B, GABARAP, GABARAPL1, and GABARAPL2 were not consistently altered in the cells from affected individuals compared to controls (Fig. 3b, d and Supplementary Figs. 3, 4). Accumulation of the lipidated form of the LC3/GABARAP subfamily proteins upon Bafilomycin A_1_ treatment was observed in the cells from the affected individuals and controls (Fig. 3c, e and Supplementary Fig. 3). Autophagic flux, measured as the difference in the lipidated form of each LC3/GABARAP subfamily protein before and after Bafilomycin A_1_ treatment^32^, was not consistently altered in the cells from affected individuals compared to controls (Fig. 3c, e and Supplementary Fig. 3). Overall, these findings suggest that autophagy induction and autophagic flux are intact in fibroblasts and lymphoblastoid cell lines from individuals with bi-allelic variants in *ATG4D*.

### Assessment of autophagy in an *ATG4D*-deficient HeLa cell model

Since differences in genetic background could mask subtle differences in autophagy, we generated an *ATG4D*-deficient HeLa cell model. The *ATG4D*-deficient HeLa cell model was confirmed to have decreased relative *ATG4D* mRNA expression and ATG4D protein levels compared to an isogenic control (Supplementary Fig. 5A, B). Analysis of autophagosome biogenesis and morphology showed comparable formation of autophagosomes between the *ATG4D*-deficient and control HeLa cells upon treatment with Torin 1 and Bafilomycin A_1_ (Supplementary Fig. 5C). In the vehicle-only treated condition, very few autophagosomes were observed (Supplementary Fig. 5C). In the Torin 1- and Bafilomycin A_1_-treated condition, autophagosomes were induced in both the *ATG4D*-deficient and control HeLa cells (Supplementary Fig. 5C). The median autophagosome area in the control HeLa cells was 7.23%, while that of the *ATG4D*-deficient HeLa cells was 7.28%; the median autophagosome size in the control HeLa cells was 0.2795 μm^2^, while that of the *ATG4D*-deficient HeLa cells was 0.2859 μm^2^ (*p* > 0.05; Supplementary Fig. 5C). Basal levels of p62 and total LC3B, GABARAPL1, and GABARAPL2 were decreased in the *ATG4D*-deficient HeLa cells compared to control (Supplementary Fig. 5D, E). Induction of the lipidated form of the LC3/GABARAP subfamily proteins upon Bafilomycin A_1_ treatment was observed in the *ATG4D*-deficient HeLa cells and control (Supplementary Fig. 5D, E). Autophagic flux was modestly decreased for GABARAP and GABARAPL1 in the *ATG4D*-deficient HeLa cells (Supplementary Fig. 5D, E).

### The p.Tyr280Cys, p.Asp356Asn, and p.Asp437Alafs*37 ATG4D variants decrease in vitro GABARAPL1 priming activity

**Fig. 4:**
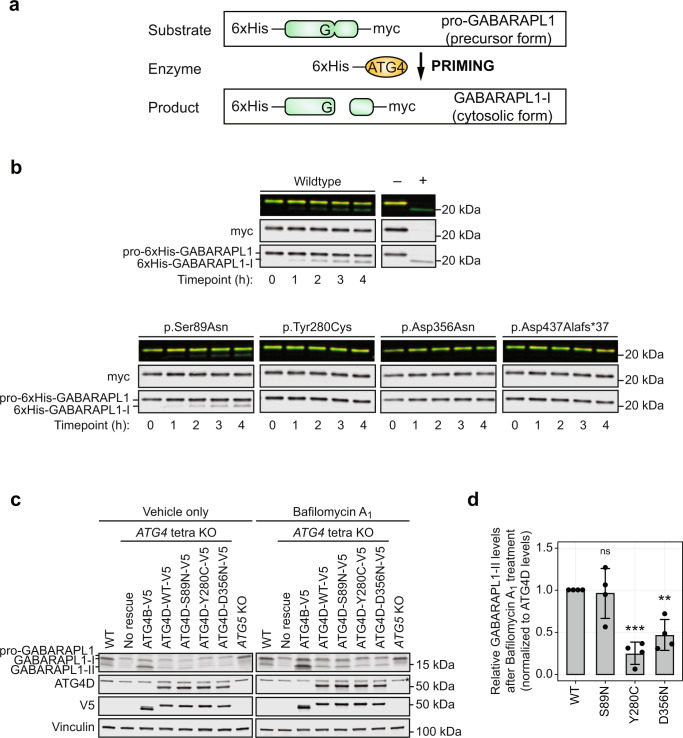
Assessment of the priming activity of the ATG4D variants using an in vitro GABARAPL1 priming assay and a GABARAPL1 priming rescue experiment. **a** A schematic of the recombinant proteins used for the in vitro GABARAPL1 priming assay. Both the pro-GABARAPL1 substrate and the N-terminally cleaved ATG4D (ΔN63 ATG4D) enzyme are tagged with 6xHis at the N-terminus for purification. The pro-GABARAPL1 substrate also has a C-terminal myc tag to visualize priming activity with increased sensitivity. **b** in vitro GABARAPL1 priming assays demonstrating the priming activity of wildtype, p.Ser89Asn, p.Tyr280Cys, p.Asp356Asn, and p.Asp437Alafs*37 ΔN63 ATG4D enzymes. Recombinant 6xHis-GABARAPL1-myc substrate was incubated with wildtype or variant ΔN63 ATG4D enzymes for the indicated time and analyzed by immunoblot. The efficient ATG4B enzyme was incubated with the G116A GABARAPL1 mutant that cannot be primed as a negative control (−) or wildtype GABARAPL1 as a positive control (+) for 1 h. Representative of *n* = 3 independent experiments. **c** GABARAPL1 priming rescue experiments demonstrating the priming activity of *ATG4* tetra knockout cells expressing V5-tagged wildtype (WT) ATG4D and the three ATG4D missense variants (p.Ser89Asn (p.S89N), p.Tyr280Cys (p.Y280C), and p.Asp356Asn (p.D356N)) treated with 200 nM Bafilomycin A_1_ or vehicle only for 8 h. *ATG4* tetra knockout cells lack the ability to prime pro-GABARAPL1 (precursor form) to GABARAPL1-I (cytosolic form) and, subsequently, also lack the ability to form GABARAPL1-II (lipidated form); this is demonstrated by the presence of only pro-GABARAPL1. *ATG5* knockout cells have the ability to prime but lack the ability to lipidate the LC3/GABARAP subfamily members; this is demonstrated by the presence of only GABARAPL1-I. Expression of V5-tagged ATG4B was used as a positive control. All targets for each treatment were assessed on the same gel. A non-specific band for the ATG4D blot is marked by an asterisk. Representative of *n* = 4 independent experiments. **d** Quantification of relative GABARAPL1-II levels after treatment with 200 nM Bafilomycin A for 8 h is represented as the mean ± standard deviation of 4 independent experiments. GABARAPL1-II levels were normalized to ATG4D levels. A one-way ANOVA was performed to compare relevant predefined dataset pairs and a Dunnett’s multiple comparisons post hoc test was performed to correct for multiple comparisons. **p < 0.01; ***p < 0.001; h, hours; kDa, kilodaltons; KO, knockout; ns, not significant; WT, wildtype.

Since the evaluation of autophagy in accessible tissues and cell types did not reveal robust defects in autophagy, possibly due to functional redundancy by the other three ATG4 cysteine protease isoforms masking the effects of the *ATG4D* variants^14^, we assessed the functional effect of each individual ATG4D variant by performing a previously described in vitro priming assay using the predominant ATG4D substrate GABARAPL1 (Fig. 4a)^15,18^. N-terminally cleaved ATG4D (ΔN63 ATG4D) has been shown to have moderate priming activity against GABARAPL1^15^, so this assay was used to measure the priming activity of each ATG4D variant. Recombinant N-terminally cleaved wildtype and variant ATG4D enzymes as well as GABARAPL1-myc substrate were expressed and purified, priming reactions were incubated for 0–4 h, and priming products were analyzed by immunoblot (Fig. 4a). While the p.Ser89Asn ATG4D variant, located upstream of the cysteine protease domain, had comparable in vitro GABARAPL1 priming activity compared to wildtype ATG4D, the p.Tyr280Cys, p.Asp356Asn, and p.Asp437Alafs*37 ATG4D protein variants displayed decreased in vitro GABARAPL1 priming activity (Fig. 4b), suggesting that these variants are loss-of-function variants and impair the priming function of ATG4D.

### The p.Tyr280Cys and p.Asp356Asn ATG4D missense variants fail to fully rescue GABARAPL1 priming activity in *ATG4* tetra knockout HeLa cells

To further investigate the functional effect of the ATG4D missense variants in a cellular system in the absence of the other three functionally redundant ATG4 isoforms, we expressed V5-tagged wildtype ATG4D or each ATG4D missense variant in a HeLa cell line with all four *ATG4* genes knocked out by CRISPR/Cas9 gene editing (Supplementary Fig. 6A)^18^. The priming and lipidation status of GABARAPL1 was assessed in the presence or absence of 200 nM Bafilomycin A_1_ for 8 h in wildtype (WT) or *ATG4* tetra knockout cells with or without rescue with each V5-tagged WT or variant ATG4D (Fig. 4c, d). *ATG4* tetra knockout cells lack the ability to prime pro-GABARAPL1 (precursor form) to GABARAPL1-I (cytosolic form) and, subsequently, also lack the ability to form GABARAPL1-II (lipidated form); this was demonstrated by the presence of only pro-GABARAPL1. *ATG5* knockout cells have the ability to prime but lack the ability to lipidate the LC3/GABARAP subfamily members; this was demonstrated by the presence of only GABARAPL1-I. In agreement with prior findings, both positive control ATG4B and WT ATG4D were able to prime GABARAPL1, as evidenced by the presence of primed GABARAPL1-I and lipidated GABARAPL1-II (Fig. 4c). The p.Ser89Asn ATG4D variant was also able to prime GABARAPL1 with a mean relative GABARAPL1-II level of 0.97 compared to WT (*p* = 0.9779, Fig. 4c, d), in keeping with the findings from the in vitro GABARAPL1 priming assay. The p.Tyr280Cys and p.Asp356Asn ATG4D variants failed to fully rescue GABARAPL1 priming with mean relative GABARAPL1-II levels of 0.24 and 0.46 compared to WT, respectively (*p* = 0.0003 and *p* = 0.0043, respectively; Fig. 4c, d). The decreased lipidated GABARAPL1-II levels for p.Tyr280Cys and p.Asp356Asn were also accompanied by higher levels of pro-GABARAPL1 (Fig. 4c). These findings further substantiate that the ATG4D missense variants located in the cysteine protease domain are loss-of-function variants and impair the priming function of ATG4D.

## Discussion

In this study, we report three individuals from two unrelated families with bi-allelic loss-of-function variants in *ATG4D* presenting with a neurodevelopmental disorder characterized by speech and motor impairment with variable disease severity and progression. An *ATG4D*-deficient HeLa cell model revealed a modest decrease in autophagy flux based on the analyses of the LC3/GABARAP subfamily members. Additionally, basal levels of total GABARAPL1 were decreased in the primary fibroblasts from all affected individuals as well as in the *ATG4D*-deficient HeLa cell line compared to the relevant controls. Furthermore, to address the functional redundancy of the other ATG4 protein isoforms in cells, a GABARAPL1 in vitro assay and GABARAPL1 priming rescue experiment assessing the priming activity of the ATG4D variants in the absence of the other ATG4 cysteine proteases in vitro or in *ATG4* tetra knockout cells demonstrated decreased in vitro GABARAPL1 priming activity for three of the four ATG4D variants and failure to completely rescue GABARAPL1 priming for two of the three ATG4D missense variants, suggesting that these variants impair the priming function of ATG4D.

Neurological impairment has been associated with ATG4D deficiency in model organisms, including those in the dog^23^, zebrafish^23^, and mouse^20^, providing additional evidence that the bi-allelic variants in *ATG4D* may underlie the neurodevelopmental disorder observed in the three affected individuals. In the canine model, genetic studies revealed a homozygous missense variant in *Atg4d* associated with cerebellar ataxia, nystagmus, and behavioral changes^23^, similar to the clinical presentation of Individual 1. Notably, incomplete penetrance and variable expressivity were observed in the canine model^23^. Cerebellar ataxia ranged from mild to severe with variable age at onset and age at euthanasia, even between littermates. Some of the affected dogs presented with nystagmus and behavioral changes while others did not, suggesting further phenotypic variability. While the majority of affected dogs presented with mild cerebellar atrophy on MRI, there were no remarkable findings for two affected dogs. These observations suggest that the variability in the clinical presentation, disease progression, and neuroimaging in the three affected individuals presented here may not be unexpected. Continued clinical follow-up will be needed to clarify disease progression for these individuals with bi-allelic variants in *ATG4D*.

The clinical spectrum and phenotypic variability between the affected individuals in Family 1 and Family 2, particularly in the clinical severity and disease course, suggests highly variable expressivity of this neurological disorder in humans, similar to what has been described in the canine model^23^. While the frequent tripping and falling, clumsiness, shuffling gait, and staring and abnormal hand movement episodes have resolved for Individual 2; the clumsiness, gait abnormalities, intention tremor, and episodic dyscoordination for Individual 1 have been slowly progressing. He additionally presents with seizures, hyporeflexia of the lower limbs, distal muscle weakness, dysarthria, nystagmus, and behavioral changes. Neuroimaging of Individual 1 revealed mild cerebellar atrophy, while the neuroimaging of Individual 2 was unremarkable (Fig. 1a). The seizures in Individual 1 may have exacerbated his clinical presentation, acting as a phenotypic modifier. Further, Individuals 1 and 2 have mild cognitive impairment, while Individual 3 is too young to assess at this time. Additionally, all three affected individuals presented with delayed speech and motor development and, interestingly, all three had a similar facial gestalt comprising almond-shaped eyes, depressed nasal bridge, and a prominent Cupid’s bow (Fig. 1b). On brain MRI, Individual 1 showed cerebellar atrophy, while Individual 2 had no remarkable findings, although Individual 2 was only 1 year and three months at the time of imaging. Additional genetic variation may contribute to the clinical presentation of Individual 1. Specifically, we identified rare, likely pathogenic compound heterozygous variants in *PRRC2A* (Supplementary Table 3), encoding for the *N*-methyladenosine (m^6^A) RNA modification reader that controls oligodendrocyte progenitor cell proliferation and fate determination^33^. While the gene has not been associated with any human disease, a conditional brain-specific murine knockout of *Prrc2a* was found to lead to hypomyelination, locomotive and cognitive defects, and decreased lifespan by affecting oligodendroglial specification^33^. While there was no evidence of hypomyelination and normal conduction velocities for age were observed in Individual 1, the potential genetic contribution of these variants cannot be overlooked. More individuals will be required to carefully evaluate the variable expressivity, genotype–phenotype correlation, and completely characterize the full spectrum of clinical features and disease progression of this neurological disorder.

Other monogenic disorders of autophagy affecting the core ATG proteins include spinocerebellar ataxia 25 (MIM 617584), which is characterized by ataxia and developmental delay due to a homozygous loss-of-function missense variant in *ATG5*^34^, and spinocerebellar ataxia 31 (MIM 619422), which is a multisystemic disorder characterized by ataxia, developmental delay, musculoskeletal abnormalities, and dysmorphic features due to bi-allelic loss-of-function variants in *ATG7*^35^. While the affected individuals reported here presented with milder neurodevelopmental disease, there is some phenotypic overlap including the developmental delay observed in all three individuals and the cerebellar signs and MRI findings in Individual 1. Interestingly, the cohort of individuals deficient for ATG7 demonstrated a highly variable clinical spectrum^35^, which also appears to be evident in our small cohort; the phenotypic variability contributes to the challenge of characterizing these disorders of autophagy dysregulation.

The functional assessment of ATG4D has been challenging due to the redundant priming and delipidating function between the four ATG4 protein isoforms^14^. To gain insight into the common and unique contributions of each ATG4 cysteine protease, several experimental approaches have been used including the re-introduction of the ATG4 isoform of interest into an *ATG4* tetra knockout cell line generated by gene editing and/or knockdown strategies for all four ATG4s^13,18^ as well as in vitro priming or delipidation assays that assess the activity of each individual ATG4 in the absence of the other three isoforms^13,15,16^. Collectively, these studies have demonstrated that ATG4D mainly primes GABARAPL1^15,18^ and delipidates the LC3/GABARAP subfamily proteins with similar enzymatic activities to the other ATG4s^13^. Our studies also confirmed GABARAPL1 as the predominant substrate for ATG4D and that the two missense variants and frameshift variant located in and downstream of the cysteine protease domain, respectively, had decreased GABARAPL1 priming activity through an in vitro assay and cellular rescue experiment (Fig. 4 and Supplementary Fig. 6), confirming that these variants are pathogenic loss-of-function variants.

Cellular effects of ATG4D deficiency in model organisms have also been investigated: increased basal lipidated LC3B protein levels were observed in affected canine fibroblasts^36^, and increased lipidated LC3/GABARAP protein levels and altered autophagosome number and size in knockout murine fibroblasts^20^. In our study, basal lipidated LC3/GABARAP protein levels were not consistently increased and autophagosomes were not altered in number or size in patient cells or in the ATG4D-deficient HeLa cell model (Figs. 2, 3 and Supplementary Figs. 3, 4 and 6). Interestingly, the authors of the knockout mouse model concluded that ATG4D is the main LC3/GABARAP delipidating enzyme^20^, which is in contrast to in vitro delipidation studies that suggest all four ATG4s contribute to delipidating the LC3/GABARAP subfamily proteins^13,17^. Altogether, these contradictory observations might arise from differences in experimental design, the nature of the *ATG4D* genetic alteration, genetic modifiers, and/or species-specific differences. Further studies are required to further delineate ATG4D function and reconcile these divergent findings.

Several studies have provided insights into the biological functions of ATG4D, particularly its autophagy-independent roles^13,15,18–20^ and contributions toward non-canonical autophagy^17^, that may be critical for further understanding disease pathogenesis. One unique aspect of ATG4D biology is its connection with the mitochondrion: ATG4D contains a cryptic mitochondrial signal peptide downstream of a caspase cleavage site (Fig. 1e) and cleavage by caspase 3 allows the N-terminally cleaved ATG4D (ΔN63 ATG4D) to localize to the mitochondria^15^. Complementary to these studies, ΔN63 ATG4D has been shown to bind liposomes containing the mitochondria-specific lipid cardiolipin^13^. Recently, a role for ATG4D in non-canonical autophagy has been uncovered^17^. Non-canonical autophagy involves the conjugation of ATG8 to single membranes (CASM) at endolysosomal compartments, leading to the conjugation of the LC3/GABARAP subfamily proteins (ATG8s) to phosphatidylserine (PS). Interestingly, ATG4D was the most efficient isoform for delipidating LC3/GABARAP-PS among the ATG4 isoforms and the only isoform to preferentially delipidate LC3/GABARAP-PS over LC3/GABARAP-PE^17^. Further, similar to ATG4A and ATG4B, ATG4D has been found to stabilize GABARAP and GABARAPL1^18,19^. Finally, ATG4D may have an indirect role in GABA receptor trafficking in the cerebellum^20,37^, which could contribute to the neuropathology observed in the *Atg4d* knockout mouse. These new aspects of ATG4D biology will guide future studies to understand disease pathogenesis.

The key limitations of this study include our current incomplete understanding of ATG4D biology, the lack of clarity on the functional pathogenicity of the p.Ser89Asn ATG4D variant, and the limited number of patients evaluated. First, the assessment of autophagy in accessible tissues and cells did not reveal any apparent defects in autophagy induction, potentially due to the redundancy of the ATG4 protein isoforms^13,14,18^, and the likelihood that ATG4D has nuanced roles in autophagy and potentially more prominent roles in other pathways, such as non-canonical autophagy^17^. Indeed, gene editing alone or in combination with knockdown strategies of all four ATG4s has been required to further elucidate the common and unique contributions of each ATG4 cysteine protease^13,14,18^. Since ATG4A, ATG4C, and ATG4D are predominantly delipidation enzymes in in vitro studies and ATG4D preferentially delipidates phosphatidylserine (PS)-conjugated LC3/GABARAP subfamily members^13,17^, it would be worthwhile to investigate this aspect of ATG4D function in future studies using isogenic controls in order to interpret biological consequences with higher sensitivity. Second, one of the alleles of Individual 1 (c.266G>A, p.Ser89Asn), while bioinformatically predicted to be damaging and in trans with a deleterious variant, did not reveal a defect in GABARAPL1 priming activity, likely due to the fact that the variant is not located in the cysteine protease domain (Fig. 1e). Further studies on ATG4D biology and its putative autophagy-independent functions, particularly on its connection with the mitochondria, will guide future experimental design. Finally, only three individuals from two unrelated families were identified. The identification of additional individuals would be invaluable in characterizing the phenotypic spectrum of ATG4D-related disease.

In summary, we have identified *ATG4D* as a candidate gene for a syndromic neurodevelopmental disorder. Prior association of ATG4D with neurodevelopmental phenotypes in model organisms further supports a role for ATG4D in neurogenetic disease. The identification of additional probands and detailed biochemical and molecular studies will help characterize the full phenotypic spectrum of disease and underlying pathophysiology.

## Methods

### Patient enrollment and consent

Individual 1 (Family 1: II-2) was evaluated through the National Institutes of Health (NIH) Undiagnosed Diseases Program (UDP)^38,39^ and was enrolled in the protocol 76-HG-0238, approved by the National Human Genome Research Institute Institutional Review Board. His mother provided written informed consent. Individual 2 (Family 2: II-3) and her younger affected sister Individual 3 (Family 2: II-4) were clinically assessed and followed through the Provincial Medical Genetics Program at the British Columbia Women’s and Children’s Hospital (Vancouver, BC, Canada). Individual 2 (Family 2: II-3), her affected younger sister Individual 3 (Family 2: II-4), two unaffected siblings (Family 2: II-1 and Family 2: II-2), and mother (Family 2: I-2) were enrolled in the protocol 76-HG-0238, approved by the National Human Genome Research Institute Institutional Review Board for sample collection and molecular analyses. Their mother provided written informed consent for all individuals in this family. The authors affirm that human research participants provided informed consent for the publication of the images in Fig. 1.

### Exome and/or genome sequencing analysis

Peripheral whole blood samples were collected, and DNA was extracted using CLIA-approved methods. Additionally, for Family 2, DNA samples were collected from several family members using the iSWAB Discovery Collection Kit (Mawi DNA Technologies, Hayward, CA) and the genomic DNA was extracted from the buccal swabs using the QIAamp DNA Mini Kit (Qiagen, Germantown, MD) for Sanger sequencing validation.

Initial trio clinical exome sequencing was performed on Individual 1 and his parents at GeneDx (Gaithersburg, MD) followed by quartet clinical genome sequencing at HudsonAlpha Genome Sequencing Center (Huntsville, AL) through the Undiagnosed Diseases Network^25–28^; genome sequencing is available in dbGaP (accession number: phs001232.v1.p1). Trio clinical exome sequencing was performed on Individual 2 and her parents at Blueprint Genetics (Seattle, WA; https://blueprintgenetics.com/). GeneMatcher (https://genematcher.org/), an online tool for connecting researchers and/or clinicians with an interest in the same gene^24^, was used to facilitate collaboration.

Research re-analysis of data from Individual 1 and his family was done through the NIH Undiagnosed Diseases Program (UDP). Briefly, sequencing reads were filtered for quality and aligned to human reference genome NCBI build 37 (hg19) using a pipeline developed by the NIH UDP, one based on NovoAlign (Novocraft Technologies, Petaling Jaya, Malaysia), and separately, a diploid aligner^40^ that was run on a commercial platform (Appistry Inc., St. Louis, MO). Variants were called with HaplotypeCaller and GenotypeGVCFs^41–43^. Variants were annotated using snpEff^44^ and a combination of publicly available data sources (gnomAD, ESP, and 1000Genomes) and internal cohort statistics. These annotations were utilized to create a list of rare, non-synonymous, start-gain/loss, stop-gain/loss, frameshift, canonical splice site variants, and intronic variants (±20 bp) that were consistent with homozygous recessive, compound heterozygous, X-linked or *de novo* dominant disease models. These variants were manually inspected using the Integrative Genomics Viewer (IGV) and checked for publicly available clinical or functional data in OMIM, HGMD, and PubMed. Variants were interpreted and prioritized based on the clinical relevance of the gene and the pathogenicity of the variants using the ACMG-AMP guidelines^45^. In the absence of candidate variants with unambiguous clinical relevance such as those in the majority of UDP cases and in this case in particular, variants were prioritized by inferred significance based on Mendelian consistency, population frequency, and predicted pathogenicity, coalesced with published biological and functional data of the genes.

All candidate variants were validated by Sanger sequencing using the primers indicated in Supplementary Table 4 on genomic DNA from peripheral blood (Family 1) or buccal swabs (Family 2). The Multiplex PCR Kit (206145, Qiagen, Germantown, MD) was used to conduct PCR amplification using the following conditions: 1 cycle of 95 °C for 15 min; 35 cycles of 94 °C for 30 s, 53 °C (Family 1) or 57 °C (Family 2) for 1 min 30 sec, and 72 °C for 1 min; followed by 1 cycle of 72 °C for 10 min. Excess primers and unincorporated nucleotides were enzymatically removed using the ExoSAP-IT PCR Product Cleanup Reagent (Applied Biosystems/Thermo Fisher Scientific, Foster City, CA) according to the manufacturer’s specifications. Sequencing was performed by Macrogen (Rockville, MD) and sequences were analyzed using Sequencher (version 5.4.6—Build 46289, Gene Codes, Ann Arbor, MI).

### Isolation and culture of patient cells

Primary fibroblasts from affected Individuals 1, 2, and 3 were cultured from a forearm skin biopsy. Unaffected primary fibroblasts GM00969 (2-year-old Caucasian female), GM01652 (11-year-old Caucasian female), and GM09503 (10-year-old Caucasian male) (Coriell Institute for Medical Research, Camden, NJ) and ATCC60235894 (neonatal male) (American Type Culture Collection, Manassas, VA) were used as unaffected controls. Fibroblasts were cultured in high glucose DMEM (11965092, Gibco/Thermo Fisher Scientific, Gaithersburg, MD) with 10% fetal bovine serum (FBS, 10082, Gibco/Thermo Fisher Scientific, Gaithersburg, MD), and 1× antibiotic-antimycotic (15240, Gibco/Thermo Fisher Scientific, Gaithersburg, MD) at 37 °C with 5% CO_2_.

Lymphoblastoid cell lines were established from the peripheral blood samples of Individual 2, her affected younger sister Individual 3, and her mother by transformation with Epstein-Barr virus (EBV)-containing supernatant through the Tissue Culture and Biobanking Shared Resource (TCBSR) at Georgetown Lombardi Comprehensive Cancer Center (Washington, DC) using standard methods^46^. Commercially available unaffected lymphoblastoid cell line E. H. IV (Elaine IV) (ATCC CCL-104, American Type Culture Collection, Manassas, VA) and the lymphoblastoid cell line established from the mother’s peripheral blood sample were used as unaffected controls. Lymphoblastoid cells were cultured in RPMI 1640 Medium with GlutaMAX Supplement (61870036, Gibco/Thermo Fisher Scientific, Gaithersburg, MD) containing 10% FBS (10082, Gibco/Thermo Fisher Scientific, Gaithersburg, MD), and 1× antibiotic-antimycotic (15240, Gibco/Thermo Fisher Scientific, Gaithersburg, MD) at 37 °C with 5% CO_2_.

### RNA extraction and reverse transcription

Cells were homogenized using the QIAshredder (79654, Qiagen, Germantown, MD) and total RNA was extracted using the RNeasy Mini Kit (74106, Qiagen, Germantown, MD). Genomic DNA was removed by on-column DNase I digestion (79254, Qiagen, Germantown, MD).

Reverse transcription was performed using the Omniscript Reverse Transcription Kit (205111, Qiagen, Germantown, MD) using up to 2 μg total RNA per 20 μl reaction and 1 μM Oligo-dT primer (O4387-.1 ML, Sigma-Aldrich, St. Louis, MO) according to the manufacturer’s specifications.

### Gene expression analysis

TaqMan Gene Expression Master Mix (4369016, Applied Biosystems/Thermo Fisher Scientific, Foster City, CA) was used with the 7500 Fast Real-Time PCR System (Applied Biosystems/Thermo Fisher Scientific, Foster City, CA) for gene expression analysis. The following conditions were used for amplification: 1 cycle of 50 °C for 2 min for uracil-N-glycosylase incubation, followed by 1 cycle of 95 °C for 10 min for DNA polymerase activation, followed by 40 cycles of 95 °C for 15 sec and 60 °C for 1 min for PCR amplification.

The relative quantification of gene expression was calculated using the delta-delta C_t_ method^47^ using the 7500 Software version 2.3 (Applied Biosystems/Thermo Fisher Scientific, Foster City, CA). Expression of *HPRT1* and *POLR2A* was used as the internal controls. All TaqMan assays used for gene expression analysis are presented in Supplementary Table 5.

### Immunoblot analysis

Cells were lysed in RIPA Buffer (R0278, Sigma-Aldrich, St. Louis, MO) containing 1× Complete Ultra Protease Inhibitor Cocktail (05892970001, Roche/Sigma-Aldrich, St. Louis, MO) for 15 min on ice. The cysteine protease inhibitor *N*-Ethylmaleimide (E3876, Sigma-Aldrich, St. Louis, MO) was added to 20 mM to stabilize the lipidated form of the LC3/GABARAP subfamily members by inhibiting the processing activity of ATG4 cysteine proteases^14^. The samples were homogenized by sonication (Model 250 Digital Sonifier, Branson Ultrasonics, Danbury, CT) at 10% amplitude for 30 s on ice. Laemmli Sample Buffer (Bio-Rad Laboratories, Hercules, CA) was added, and samples were incubated at 95 °C for 5 min. Samples were resolved on a 7.5% (for ATG4D) or 15% (for in vitro GABARAPL1 priming assay) or 4–15% or 4–20% gradient (for all other targets) polyacrylamide gel and transferred to a PVDF membrane (IPFl00010, Millipore, Burlington, MA). Membranes were blocked using Odyssey Blocking Buffer (LI-COR Biosciences, Lincoln, NE) for 1 h at room temperature and subsequently incubated with primary antibodies diluted in Odyssey Blocking Buffer with 0.1% Tween 20 overnight at 4 °C. After four 5-min washes with TBS-T (0.1% Tween 20 in Tris-buffered saline), membranes were incubated with IRDye-conjugated secondary antibodies (1:10,000, LI-COR Biosciences, Lincoln, NE) for 1 h at room temperature. After four 5-min washes with TBS-T, two 5-min washes with TBS were performed to remove residual Tween 20. Membranes were imaged on the Odyssey CLx Infrared Imaging System and analyzed using the CLx Image Studio version 3.1 software (LI-COR Biosciences, Lincoln, NE). All blots were derived from the same experiment and were processed in parallel. All primary antibodies and dilutions used for immunoblotting are presented in Supplementary Table 6. All uncropped images of the data presented in Figs. 3, 4 are presented in Supplementary Figs. 7–10.

### Immunoblot analysis to confirm total basal GABARAPL1 protein levels

Primary fibroblasts were treated with vehicle or Bafilomycin A_1_ for 3 h. Cell pellets were lysed using RIPA Lysis Buffer (sc-24948, Santa Cruz Biotechnology) supplemented with Complete Mini Protease Inhibitor Cocktail (11836153001, Roche). The cysteine protease inhibitor *N*-Ethylmaleimide (E3876, Sigma-Aldrich), which has been shown to inhibit ATG4 activity and stabilize lipidated GABARAP and GABARAPL1^14^, was added to a final concentration of 20 mM. The Pierce BCA Protein Assay Kit (23225, Thermo Scientific) was used for total protein quantification, and 20 μg of protein was loaded on a 4–12% gradient Bolt Bis-Tris gel (Invitrogen) for separation. Gel was then transferred to a PVDF membrane (Bio-Rad Laboratories) and blocked with 2% milk solution before incubation overnight at 4 °C with primary antibodies. Primary antibodies were diluted with Odyssey® Blocking Buffer in PBS (LI-COR Biosciences). Membranes incubated with primary antibody were washed with 1× PBS-T (0.1% Tween 20) and incubated with the appropriate secondary antibody (goat anti-mouse IgG-horseradish peroxidase (HRP) and goat anti-rabbit IgG-HRP) (Santa Cruz Biotechnology) for 1 h. The SuperSignal™ West Femto Maximum Sensitivity Substrate (34096, Thermo Scientific) and ChemiDoc MP Imaging System (Bio-Rad Laboratories) were used to detect and visualize protein bands. Densitometry was performed using Image Lab Software (Bio-Rad Laboratories) to measure the relative protein of interest present by normalizing to loading control (β-actin).

### Generation of an *ATG4D* overexpression cell line

The MGC Human *ATG4D* Sequence-Verified cDNA (MHS6278-202833433, GE Dharmacon, Lafayette, CO) was used to amplify the *ATG4D* ORF by PCR. PCR products were then cloned into the pENTR entry vector using the pENTR/D-TOPO Cloning Kit (Invitrogen/Thermo Fisher Scientific, Carlsbad, CA). Single clones were Sanger sequenced to verify sequence integrity of the *ATG4D* ORF. LR recombination was performed using the Gateway LR Clonase II Enzyme Mix (Invitrogen/Thermo Fisher Scientific, Carlsbad, CA) to recombine the *ATG4D* ORF into the pLenti6.3/V5-DEST destination vector (V53306, Invitrogen/Thermo Fisher Scientific, Carlsbad, CA). Single clones were Sanger sequenced to verify the recombination and sequence integrity of the *ATG4D* ORF.

The ViraPower Lentiviral Expression System (Invitrogen/Thermo Fisher Scientific, Carlsbad, CA) was used to overexpress *ATG4D*. In brief, lentivirus was generated by transfecting 293FT cells with the pLenti6.3-ATG4D construct and the ViraPower Lentiviral Packaging Mix (K497500, Invitrogen/Thermo Fisher Scientific, Carlsbad, CA) using Lipofectamine 2000 Transfection Reagent (Invitrogen/Thermo Fisher Scientific, Carlsbad, CA). Primary fibroblasts were transduced with the viral supernatant and stable cell lines were selected by antibiotic selection (2 μg/ml blasticidin) for 10 days. Overexpression of *ATG4D* mRNA and ATG4D protein were confirmed by quantitative PCR and immunoblot. TaqMan gene expression assays used for quantitative PCR and antibodies used for immunoblotting are presented in Supplementary Tables 5, 6, respectively.

### Generation of an *ATG4D*-deficient HeLa cell line

CRISPR-Cas9 technology was used to generate an *ATG4D*-deficient HeLa cell line. In brief, a HeLa cell line with stable and constitutive Cas9 expression (SL503, GeneCopoeia, Rockville, MD) was transfected with three pGS-gRNA-Neo plasmids (GenScript, Piscataway, NJ) each containing a guide RNA, under the control of the U6 promoter, targeting *ATG4D* using Lipofectamine 2000 Transfection Reagent (Invitrogen/Thermo Fisher Scientific, Carlsbad, CA) according to the manufacturer’s specifications. In parallel, the phU6-gRNA plasmid (#53188, Addgene, Watertown, MA) was transfected to serve as an empty vector control. Transfected cells were selected with 500 μg/ml Geneticin (10131035, Gibco/Thermo Fisher Scientific, Gaithersburg, MD) for 10 days and were maintained in high glucose DMEM (11965092, Gibco/Thermo Fisher Scientific, Gaithersburg, MD) with 100 μg/ml Geneticin (10131035, Gibco/Thermo Fisher Scientific, Gaithersburg, MD), 10% fetal bovine serum (FBS, 10082, Gibco/Thermo Fisher Scientific, Gaithersburg, MD), and 1× antibiotic-antimycotic (15240, Gibco/Thermo Fisher Scientific, Gaithersburg, MD) at 37 °C with 5% CO_2_ thereafter. The deficiency of *ATG4D* mRNA and ATG4D protein was confirmed by quantitative PCR and by immunoblot analysis, respectively (Supplementary Fig. 5A, B). The gRNA sequences are listed in Supplementary Table 7, TaqMan assay IDs used for quantitative PCR are listed in Supplementary Table 5, and antibodies used for immunoblotting are listed in Supplementary Table 6.

### Generation of *ATG4* tetra knockout rescue HeLa cell lines

The *ATG4* tetra knockout and *ATG5* knockout HeLa cell lines were gifts from Dr. Michael Lazarou (Biomedicine Discovery Institute, Monash University, Melbourne, Australia)^18,48^. Lentiviruses were generated to express WT or each ATG4D missense variant as described above. In brief, site-directed mutagenesis was performed to introduce ATG4D missense variants in the pENTR-ATG4D plasmid using the Q5 Site-Directed Mutagenesis Kit (E0554S, New England BioLabs, Ipswich, MA) and the primers listed in Supplementary Table 4. All constructs were verified by Sanger sequencing. LR recombination was performed using the Gateway LR Clonase II Enzyme Mix (Invitrogen/Thermo Fisher Scientific, Carlsbad, CA) to recombine the wildtype and variant *ATG4D* ORFs into the pLenti6.3/V5-DEST destination vector (V53306, Invitrogen/Thermo Fisher Scientific, Carlsbad, CA). Single clones were Sanger sequenced to verify the recombination and sequence integrity of the *ATG4D* ORF. Expression of V5-tagged ATG4D WT and variant proteins after viral transduction and antibiotic selection were confirmed by immunoblot. Antibodies used for immunoblotting are presented in Supplementary Table 6.

### Transmission electron microscopy (TEM)

To assess the area and size of autophagosomes formed in response to the autophagy inducer Torin 1 and autophagy inhibitor Bafilomycin A_1_, cultured cells were treated with 100 nM of each inhibitor or vehicle (DMSO) for 3 h. Cells were then fixed using 2.5% glutaraldehyde and 1% paraformaldehyde in 0.1 M cacodylate buffer (pH 7.4) at room temperature. After 30 min, the cells were scraped from the culture plate and centrifuged at 16,000 × *g* for 5 min; fixation was continued as a cell pellet for 1 h 30 min at room temperature and for a minimum of 48 h at 4 °C thereafter. Cell pellets were washed three times with 0.1 M cacodylate buffer, fixed with 1% OsO_4_ for 2 h, washed three times with 0.1 M cacodylate buffer, washed with water, and incubated in 1% uranyl acetate for 30 min. After fixation, the samples were subsequently dehydrated, embedded, sectioned, and stained for transmission electron microscopy (TEM) as previously described^49^.

### Morphometric analysis of autophagosomes from TEM images

ImageJ was used to analyze the morphometry of autophagosomes in TEM images taken at 4,000×^50^. For each image, the cytoplasmic area was calculated by selecting the whole cell and the nucleus as regions of interest using the polygon tool, measuring the total area and the nuclear area, and then subtracting the nuclear area from the total area.

Autophagosomes were identified based on their distinctive morphology of cytoplasmic contents within double-membraned organelles^31,51^. Each autophagosome was selected as a region of interest using the polygon tool and the area was measured. Autophagosome area was assessed as the % cytoplasmic area per image and autophagosome size was assessed as the area of each individual autophagosome per sample.

### in vitro GABARAPL1 priming assay

The N-terminal 6xHis-tagged pTrcHisB-ATG4B, pTrcHisB-ΔN63 ATG4D, pTrcHisB-GABARAPL1-myc, and pTrcHisB-GABARAPL1-G116A-myc bacterial expression plasmids were a gift from Dr. Jon D. Lane (University of Bristol, United Kingdom)^15^. Site-directed mutagenesis was performed to introduce patient variants into the pTrcHisB-ΔN63 ATG4D construct using the Q5 Site-Directed Mutagenesis Kit (E0554S, New England BioLabs, Ipswich, MA) and the primers used are listed in Supplementary Table 4. To perform site-directed mutagenesis for the c.1310_1328del variant, deletion of the relevant 19 bp was performed, followed by an insertion to mimic the predicted frameshift variant. All constructs were verified by Sanger sequencing.

Recombinant proteins were expressed in BL21-CodonPlus (DE3)-RIPL Competent Cells (230280, Agilent Technologies, Santa Clara, CA). A single colony was cultured in 10 ml LB media containing 100 μg/ml carbenicillin and 50 μg/ml chloramphenicol at 37 °C and 250 rpm until the OD_600_ measured 0.5-0.6. Each culture was expanded by the addition of 1 ml of the starter culture to 50 ml of LB media without antibiotics and cultured at 37 °C and 250 rpm until the OD_600_ measured 0.5-0.6. The cultures were then cooled to room temperature and induced with 0.2 mM IPTG at 16 °C and 225 rpm for 16 h.

Recombinant proteins were purified using TALON Spin Columns (Takara Bio, Mountain View, CA). Briefly, cells were collected by centrifugation at 3500 × *g* for 20 min at 4 °C. Lysates were prepared by the addition of 10 ml xTractor Buffer (HisTALON Buffer Set, Takara Bio, Mountain View, CA) containing 2 U/ml DNase I (4716728001, MilliporeSigma, Burlington, MA), 6 mM MgCl_2_, and 1 mM CaCl_2_. For the purification of 6xHis-ATG4B, 6xHis-GABARAPL-myc, and 6xHis-GABARAPL1-G116A-myc, ProteoGuard EDTA-free Protease Inhibitor Cocktail (Takara Bio, Mountain View, CA) was added according to the manufacturer’s specifications. For the purification of 6xHis-ΔN63 ATG4D, 40 μg/ml bestatin (10874515001, MilliporeSigma, Burlington, MA), 2 μg/ml leupeptin (11017101001, MilliporeSigma, Burlington, MA), 2 μg/ml aprotinin (10236624001, MilliporeSigma, Burlington, MA), 1 μg/ml pepstatin (10253286001, MilliporeSigma, Burlington, MA), and 75 μg/ml lysozyme (L1667, MilliporeSigma, Burlington, MA) were added. Cell pellets were gently resuspended and incubated with gentle shaking for 1 h at 4 °C. Following cell lysis, samples were sonicated at 20% amplitude for 6 × 10-s pulses on ice with 10 sec cooling in between each pulse. Lysates were clarified by centrifugation at 6000 × *g* for 40 min at 4 °C. Clarified lysates were then concentrated using an Amicon Ultra-15 Centrifugal Filter Unit (UFC901024, MilliporeSigma, Burlington, MA). The TALON Spin Columns (Takara Bio, Mountain View, CA) were equilibrated, and clarified lysates were added to the column according to the manufacturer’s specifications. Following sample binding, the column was washed three times with equilibration buffer (HisTALON Buffer Set, Takara Bio, Mountain View, CA) and an intermediate wash was performed with wash buffer (10 mM imidazole in Equilibration Buffer from the HisTALON Buffer Set, Takara Bio, Mountain View, CA) prior to elution. His-tagged proteins were eluted with 2 × 600 μl elution buffer (HisTALON Buffer Set, Takara Bio, Mountain View, CA). The 6xHis-ΔN63 ATG4D recombinant proteins were concentrated using an Amicon Ultra-0.5 Centrifugal Filter Unit (UFC501096, MilliporeSigma, Burlington, MA).

To assess the GABARAPL1 priming activity of each ATG4D variant, a previously described in vitro GABARAPL1 priming assay was performed^15^. Purified recombinant proteins were incubated for 0, 1, 2, 3, or 4 h at 37 °C in HEPES buffer (0.1% CHAPS, 10% (w/v) sucrose, 5 mM DTT, 2 mM EDTA, 50 mM HEPES, pH 7.4), and reactions were stopped by the addition of Laemmli Sample Buffer (Bio-Rad Laboratories, Hercules, CA) to 1× and samples were incubated at 95 °C for 5 min and resolved on a 15% polyacrylamide gel. Priming activity was analyzed by immunoblotting using the GABARAPL1 and myc antibodies noted in Supplementary Table 6.

### GABARAPL1 priming rescue experiment

To assess the GABARAPL1 priming activity of each ATG4D missense variant, a previously described GABARAPL1 priming rescue experiment was performed^18^. In brief, *ATG4* tetra knockout cell lines expressing V5-tagged wildtype or variant ATG4D were evaluated for their ability to prime GABARAPL1 in the presence or absence of 8 h Bafilomycin A_1_ treatment by immunoblot. The presence of lipidated GABARAPL1-II, which requires the formation of primed GABARAPL1-I from pro-GABARAPL1, was considered as successful priming by an ATG4 isoform. Antibodies used for immunoblotting are presented in Supplementary Table 6.

### Statistical analyses

All statistical tests were performed using GraphPad Prism 8 Version 8.4.3. For experiments comparing two sets of data, a two-tailed Mann–Whitney *U* test was performed. For experiments involving the comparison of multiple sets of data, a one-way ANOVA or Kruskal–Wallis test was performed to compare relevant predefined dataset pairs and a Dunnett’s or Dunn’s multiple comparisons post hoc test was performed to correct for multiple comparisons, respectively. A *p*-value of less than 0.05 was considered statistically significant.

### Reporting summary

Further information on research design is available in the Nature Portfolio Reporting Summary linked to this article.

## Supplementary information

Supplementary Information

Reporting Summary Checklist

## Data Availability

Data that support the findings of this study are available from the corresponding author upon reasonable request. The ClinVar accession numbers for the variants reported in this study are VCV001322010.1, VCV001322025.1, VCV001322026.1, and VCV001801476.1. Genome sequencing data for Individual 1 is available in dbGAP, (accession number: phs001232.v1.p1). Trio clinical exome sequencing for Individual 2 and family was performed by a commercial laboratory, Blueprint Genetics (Seattle, WA; https://blueprintgenetics.com/), and the family was referred to this study as a remote collaboration. As such, we are unable to share this data. Sanger sequencing was however used to validate the variant in this family.
