# Supplementary material for: Bi-allelic ATG4D variants are associated with a neurodevelopmental disorder characterized by speech and motor impairment
Source: NPJ Genom Med. 2023 Feb 10;8:4. doi: 10.1038/s41525-022-00343-8 (PMC9918471; doi:10.1038/s41525-022-00343-8)
Supplement: Supplementary file 1 — Supplementary Information [file 41525_2022_343_MOESM1_ESM.pdf]

SUPPLEMENTARY INFORMATION

Bi-allelic *ATG4D* variants are associated with a neurodevelopmental disorder characterized by speech and motor impairment

Marie Morimoto, Vikas Bhambhani, Nour Gazzaz, Mariska Davids, Paalini Sathiyaseelan, Ellen F. Macnamara, Jennifer Lange, Anna Lehman, Patricia M. Zerfas, Jennifer L. Murphy, Maria T. Acosta, Camille Wang, Emily Alderman, Undiagnosed Diseases Network, Sara Reichert, Audrey Thurm, David R. Adams, Wendy J. Introne, Sharon M. Gorski, Cornelius F. Boerkoel, William A. Gahl, Cynthia J. Tifft, May Christine V. Malicdan

SUPPLEMENTARY FIGURES

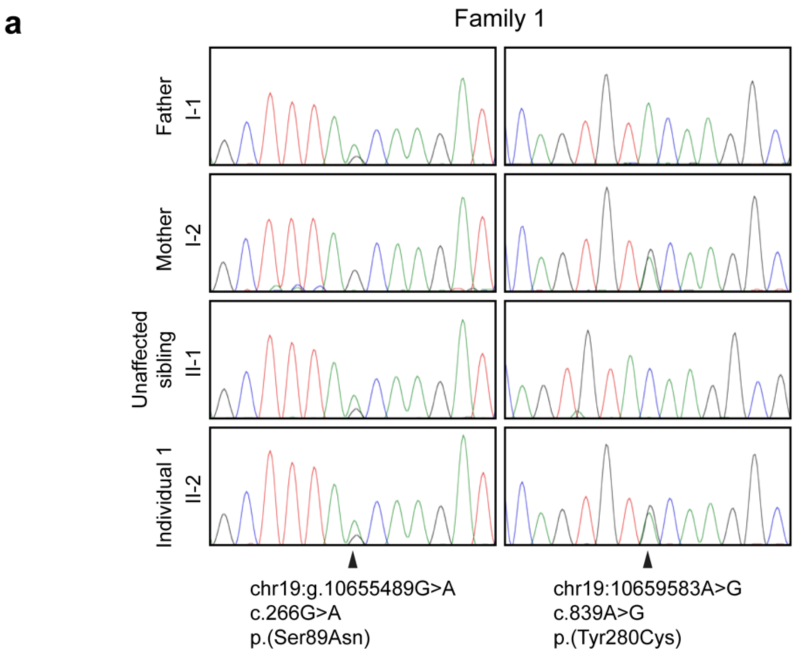

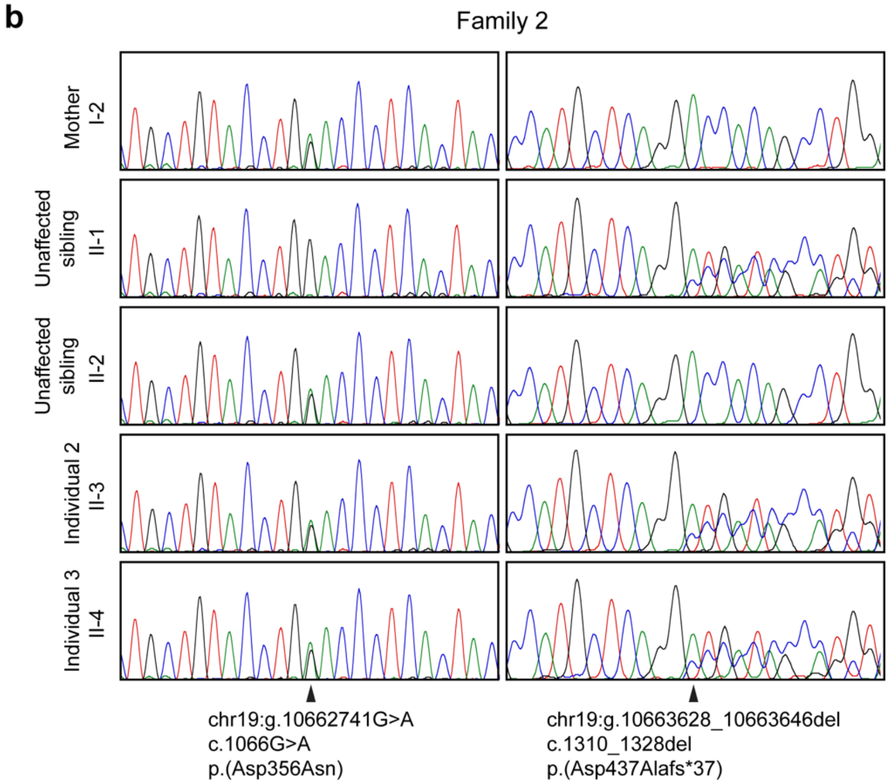

Supplementary Figure 1. Sanger sequencing chromatograms of the affected individuals with bi-allelic variants in *ATG4D* (NM_032885.5) and their family members. (A) Individual 1 has a paternally inherited c.266G>A variant (arrowhead, left panel) and a maternally inherited c.839A>G variant (arrowhead, right panel) in *ATG4D*. (B) Individual 2 and her affected sister Individual 3 both have a paternally inherited c.1310_1328del variant (arrowhead, left panel) and a maternally inherited c.1066G>A variant (arrowhead, right panel) in *ATG4D*.

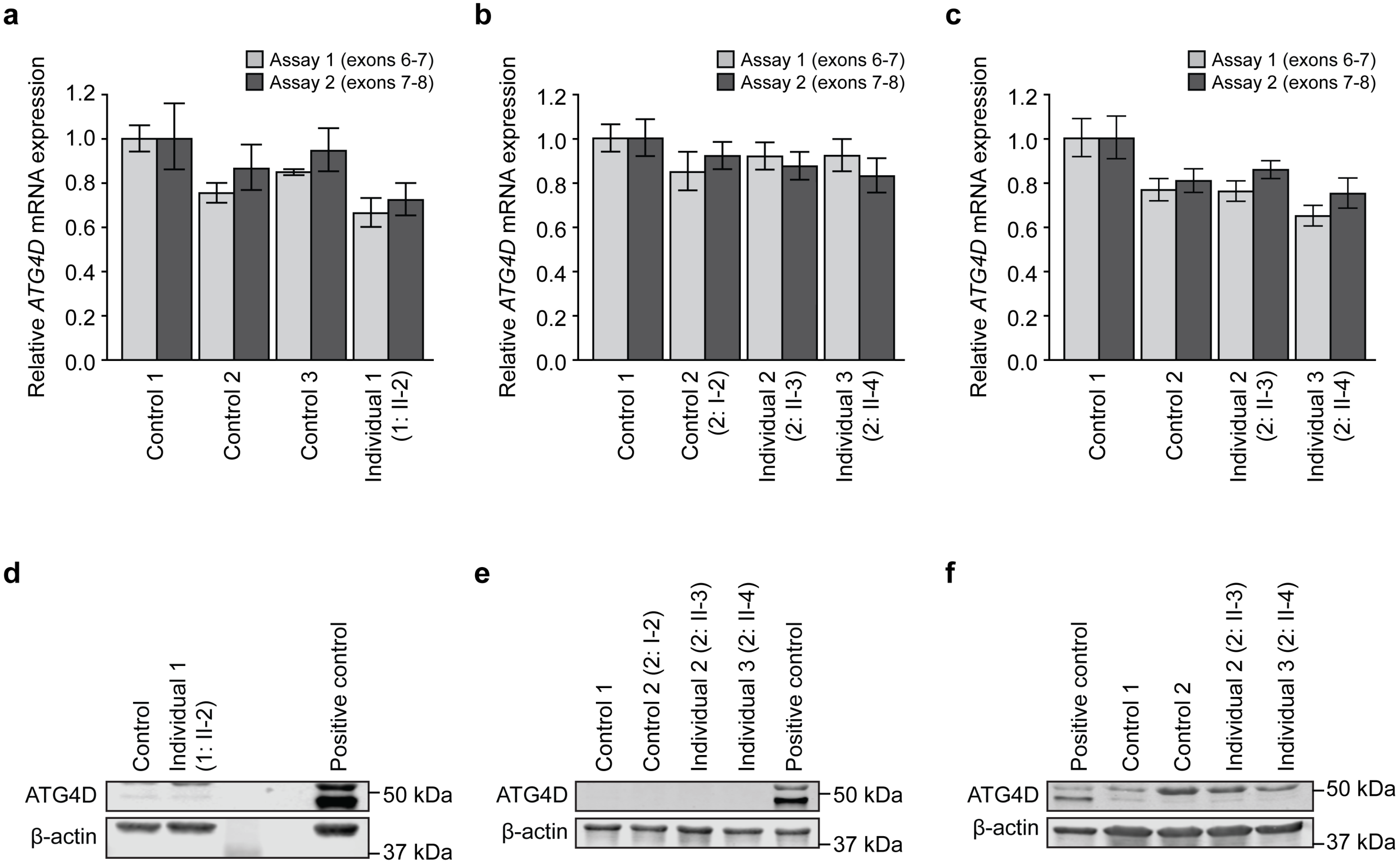

Supplementary Figure 2. Analyses of relative *ATG4D* mRNA expression and ATG4D protein levels in the cultured cells of individuals with bi-allelic variants in *ATG4D*. (A-C) Relative *ATG4D* mRNA expression was quantified by TaqMan qPCR assay in the primary fibroblasts of Control 1 (ATCC60235894), Control 2 (GM01652), Control 3 (GM09503), and Individual 1 (Family 1: II-

2) (A); the lymphoblastoid cell lines of Control 1 (CCL-104), Control 2 (Family 2: I-2, mother of Individuals 2 and 3), Individual 2 (Family 2: II-3), and Individual 3 (Family 2: II-4) (B); and the primary fibroblasts of Control 1 (ATCC60235894), Control 2 (GM00969), Individual 2 (Family 2: II-3), and Individual 3 (Family 2: II-4) (C). The data are presented as the mean of 3 technical replicates relative to the relevant Control 1. Expression of *HPRT1* and *POLR2A* were used as internal controls to normalize gene expression; error bars represent one standard deviation. (D-F) ATG4D protein levels were assessed by immunoblot analysis in the primary fibroblasts of Control 1(GM09503) and Individual 1 (D); the lymphoblastoid cell lines of Control 1 (CCL-104), Control 2 (Family 2: I-2, mother of Individuals 2 and 3), Individual 2 (Family 2: II-3), and Individual 3 (Family 2: II-4) (E); and the primary fibroblasts of Control 1 (ATCC60235894), Control 2 (GM00969), Individual 2 (Family 2: II-3), and Individual 3 (Family 2: II-4) (F). Lysate extracted from fibroblasts overexpressing ATG4D was used as a positive control and β-actin was used as a loading control.

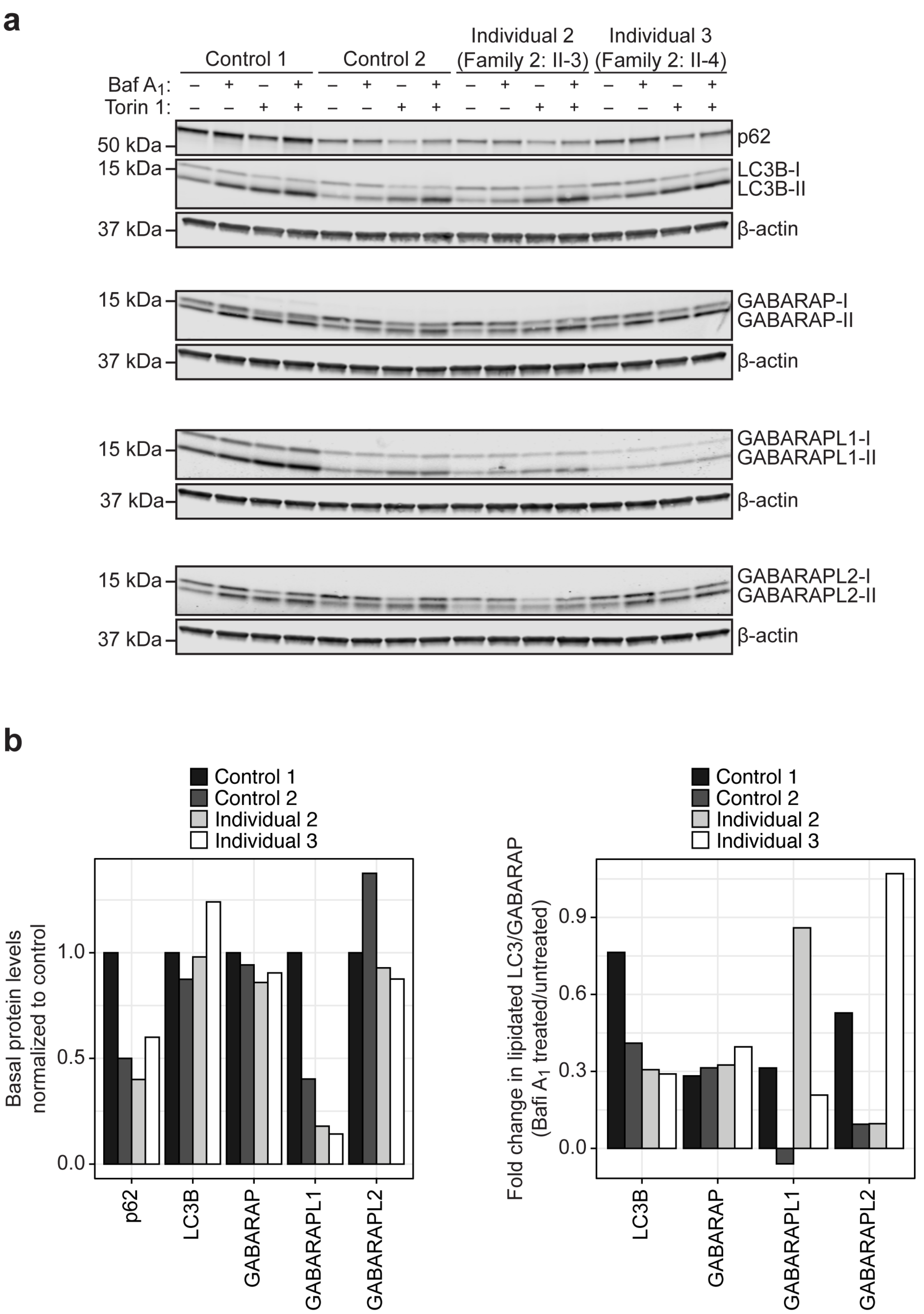
a
Control 1
Control 2
Individual 2 (Family 2: II-3)
Individual 3 (Family 2: II-4)
Baf A_1:
Torin 1:
50 kDa
15 kDa
37 kDa
p62
LC3B-I
LC3B-II
β-actin
GABARAP-I
GABARAP-II
GABARAPL1-I
GABARAPL1-II
GABARAPL2-I
GABARAPL2-II
b
Control 1
Control 2
Individual 2
Individual 3
Basal protein levels normalized to control
0.0
0.5
1.0
p62
LC3B
GABARAP
GABARAPL1
GABARAPL2
Fold change in lipidated LC3/GABARAP (Bafi A_1 treated/untreated)
0.0
0.3
0.6
0.9

Supplementary Figure 3. Analysis of the induction of lipidated LC3/GABARAP subfamily proteins in cultured cells from individuals with bi-allelic variants in *ATG4D*. (A) Immunoblot analysis of primary fibroblasts from Control 1 (ATCC60235894), Control 2 (GM00969), Individual 2 (Family 2: II-3), and Individual 3 (Family 2: II-4) assessing p62, LC3B, GABARAP, GABARAPL1, and GABARAPL2 upon induction and/or inhibition of autophagy by treatment with 100 nM Torin 1 and/or 100 nM Bafilomycin A_1 for 3 h. β-actin was used as a loading control. p62 and LC3B were assessed on the same gel. (B) Quantification of basal protein levels (left) and autophagic flux (right) from immunoblot analyses of p62, total LC3B, total GABARAP, total GABARAPL1, and total GABARAPL2 in primary fibroblasts from Control 1 (ATCC60235894), Control 2 (GM00969), Individual 2 (Family 2: II-3), and Individual 3 (Family 2: II-4). Autophagic flux is presented as the fold change of the lipidated form of each LC3/GABARAP subfamily member after treatment with Bafilomycin A_1 compared to before treatment. Abbreviation: Baf A_1, Bafilomycin A_1, kDa, kilodaltons.

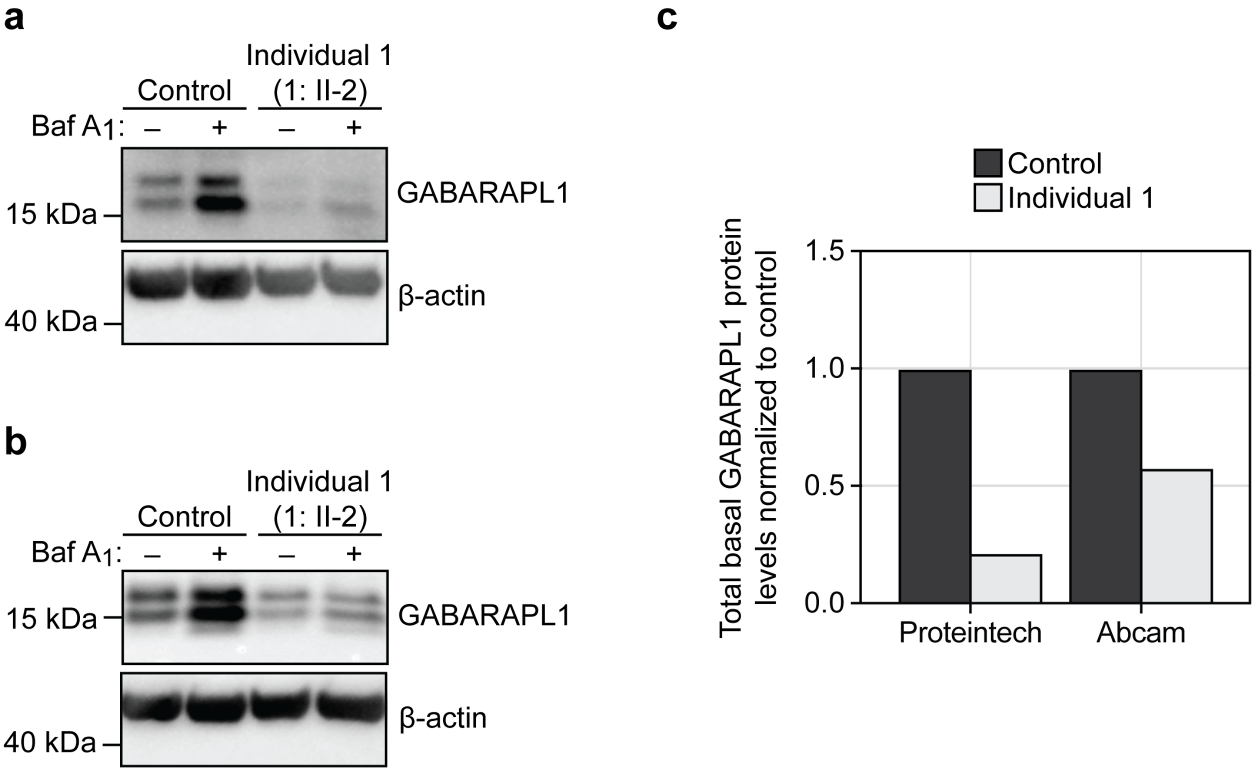

Supplementary Figure 4. Confirmation of decreased total basal GABARAPL1 protein levels in the primary fibroblasts of Individual 1 compared to an unaffected control. (A and B) Immunoblot analysis of GABARAPL1 and β-actin (loading control) in Control (GM09503) and Individual 1 (Family 1: II-2) with and without Bafilomycin A_1 (Baf A_1) treatment. β-actin was used as a loading control. Two different GABARAPL1 primary antibodies were used to confirm the decreased total basal GABARAPL1 protein levels: Proteintech 11010-1-AP in (A) and Abcam ab229558 in (B). (C) Quantification of the target of interest (cytosolic and lipidated form of GABARAPL1) normalized to the β-actin loading control and then normalized to the Control for each primary antibody. Abbreviation: Baf A_1, Bafilomycin A_1.

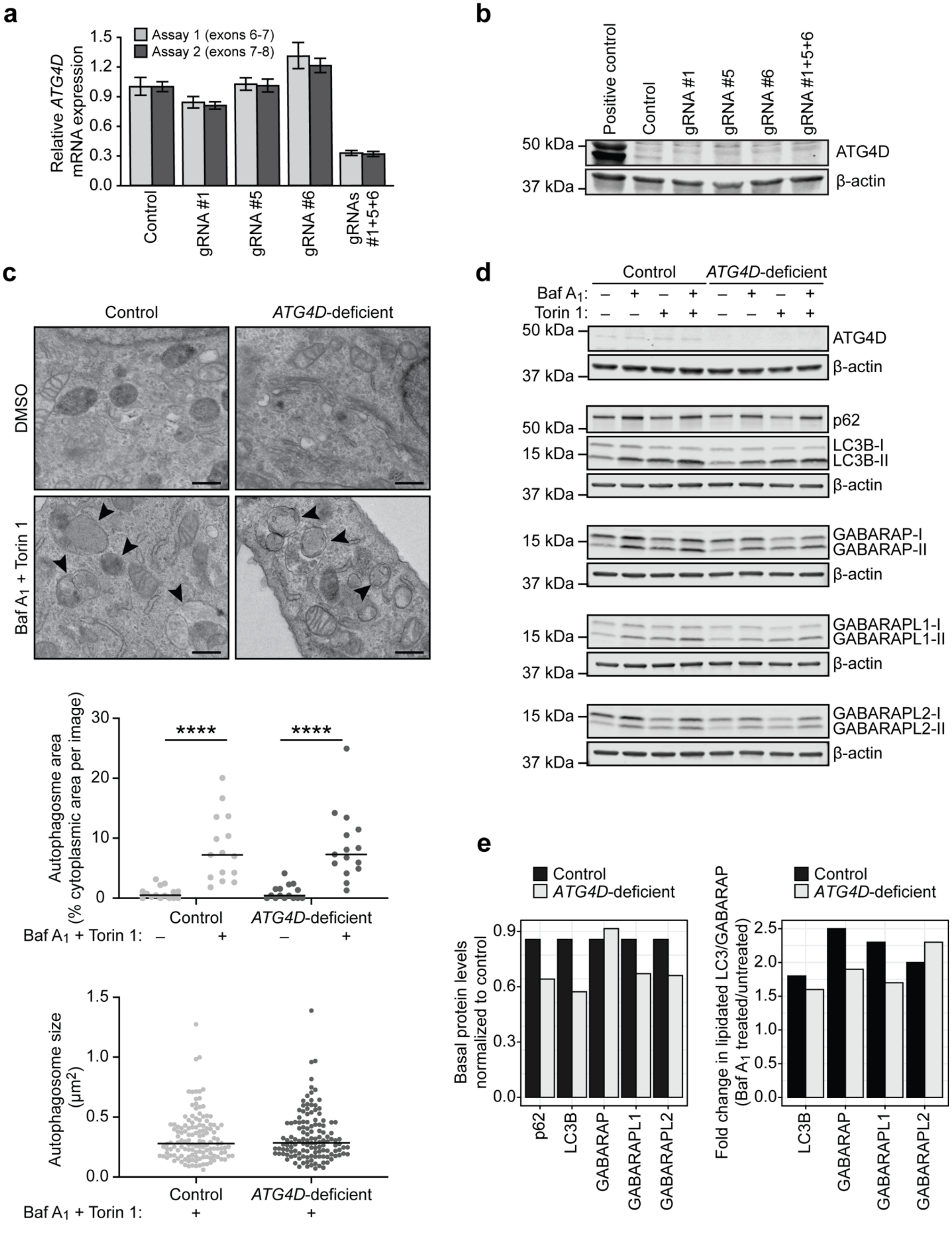

Supplementary Figure 5. Analysis of autophagosome biogenesis and induction of lipidated LC3/GABARAP subfamily proteins in an *ATG4D*-deficient HeLa cell line. *ATG4D*-deficient

HeLa cells were generated by CRISPR-Cas9 technology using one or more gRNA expression plasmids targeting *ATG4D*. (A) Relative *ATG4D* mRNA expression was quantified by TaqMan assay. The data are presented as the mean of 3 technical replicates relative to the empty vector control. Expression of *HPRT1* and *POLR2A* were used as internal controls to normalize gene expression; error bars represent one standard deviation. (B) ATG4D protein levels were assessed by immunoblot analysis. Lysate extracted from fibroblasts overexpressing ATG4D was used as a positive control and β-actin was used as a loading control. (C) Representative TEM images of control and the *ATG4D*-deficient HeLa cell line generated using all 3 gRNA plasmids treated with vehicle (DMSO) or 100 nM Torin 1 and 100 nM Bafilomycin A_1 for 3 h to induce the formation and prevent the lysosomal degradation of autophagsosomes, respectively (arrowheads). Magnification: 4000×. Scale bar: 500 nm. Quantification of the autophagosome area and size from the experiment represented in the upper panel. The data are represented as dot plots with the median indicated by a horizontal line. Light grey data points represent measurements made on empty vector control HeLa cells; dark grey data points represent measurements made on *ATG4D*-deficient HeLa cells. For the quantification of autophagosome area, 15 images taken from each sample were assessed per condition. A Kruskal-Wallis test and Dunn's multiple comparisons test were performed for relevant predefined dataset pairs. For the quantification of autophagosome size, data points represent individual autophagosomes measured from Torin 1- and Bafilomycin A_1-treated conditions. A total of $n = 134$ and $n = 130$ autophagosomes were respectively measured for control and *ATG4D*-deficient HeLa cells, and a two-tailed Mann-Whitney U test was performed. Only statistically significant comparisons are shown. (D) Immunoblot analysis of the *ATG4D*-deficient HeLa cell line assessing p62, LC3B, GABARAP, GABARAPL1, and GABARAPL2 upon induction and/or inhibition of autophagy by treatment with 100 nM Torin 1 and/or 100 nM Bafilomycin A_1 for 3 h. β-actin was used as a loading control. p62 and LC3B were assessed on the same gel. (E) Quantification of basal protein levels (left) and autophagic flux (right) from immunoblot analyses of p62, total LC3B, total GABARAP, total GABARAPL1, and total GABARAPL2 in Control and *ATG4D*-deficient HeLa cell lines. Autophagic flux is presented as the fold change of the lipidated form of each LC3/GABARAP subfamily member after treatment with Bafilomycin A_1 compared to before treatment. Abbreviations: ****, $p < 0.0001$; Baf A_1, Bafilomycin A_1; DMSO, dimethyl sulfoxide; kDa, kilodaltons; TEM, transmission electron microscopy.

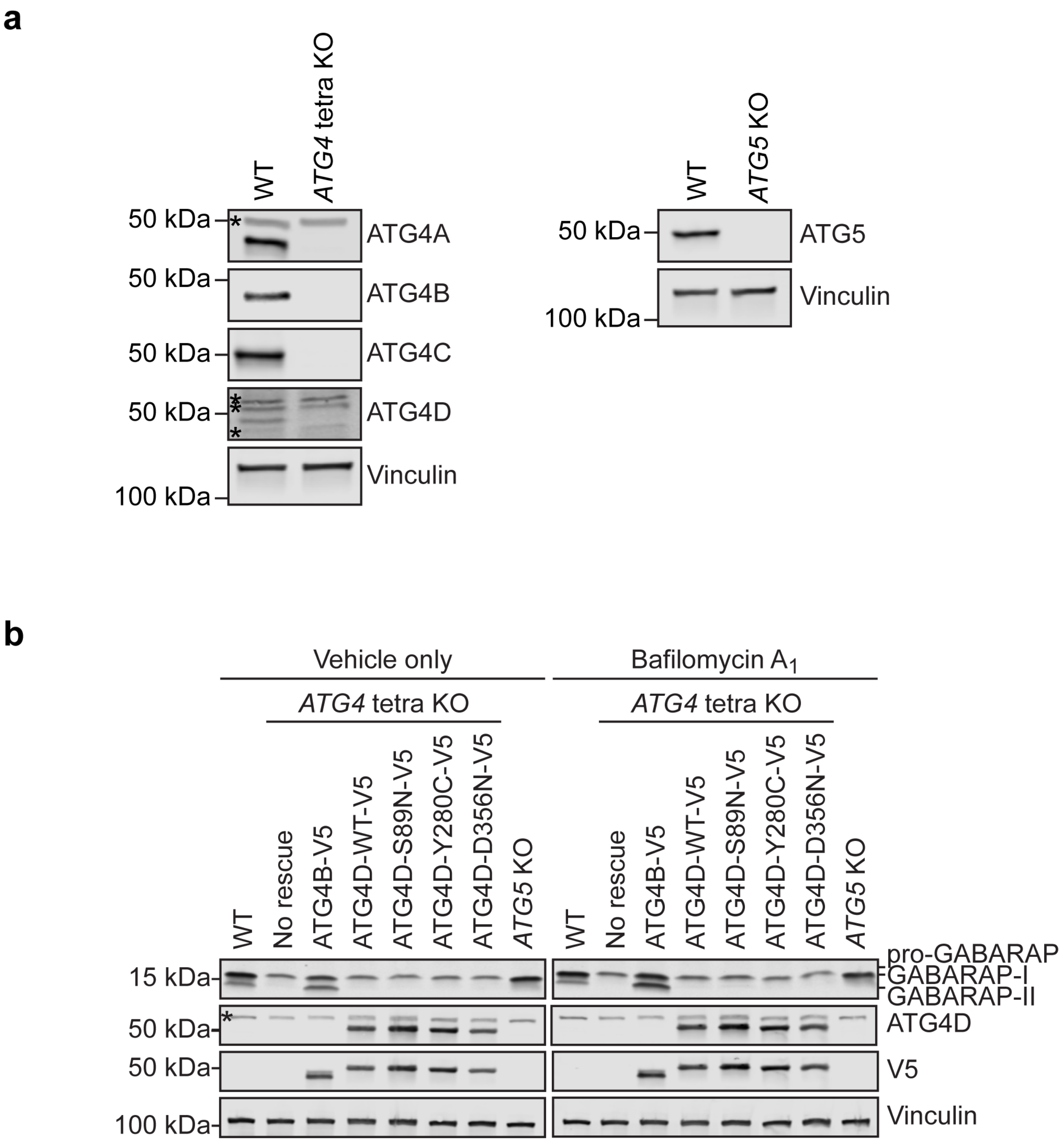

Supplementary Figure 6. *ATG4* knockout and *ATG5* knockout HeLa cell line validation and a GABARAP priming rescue experiment. (A) WT, *ATG4* tetra knockout, *ATG5* knockout were validated using immunoblotting. Vinculin was used as a loading control. All targets were assessed on different gels (a representative blot for vinculin is shown) and non-specific bands for the ATG4A and ATG4D blots are marked by asterisks. (B) GABARAP priming rescue experiments demonstrating the priming activity of *ATG4* tetra knockout cells expressing V5-tagged wildtype (WT) ATG4D and the three ATG4D missense variants (p.Ser89Asn (p.S89N), p.Tyr280Cys (p.Y280C), and p.Asp356Asn (p.D356N)) treated with 200 nM Bafilomycin A_1 or

vehicle only for 8 h. *ATG4* tetra knockout cells lack the ability to prime pro-GABARAP (precursor form) to GABARAP-I (cytosolic form) and, subsequently, also lack the ability to form GABARAP-II (lipidated form); this is demonstrated by the presence of only pro-GABARAP. *ATG5* knockout cells have the ability to prime but lack the ability to lipidate the LC3/GABARAP subfamily members; this is demonstrated by the presence of only GABARAP-I. Expression of V5-tagged ATG4B was used as a positive control. All targets for each treatment were assessed on the same gel. A non-specific band for the ATG4D blot is marked by an asterisk. Representative of n = 4 independent experiments. Abbreviations: kDa, kilodalton; KO, knockout; WT, wildtype.

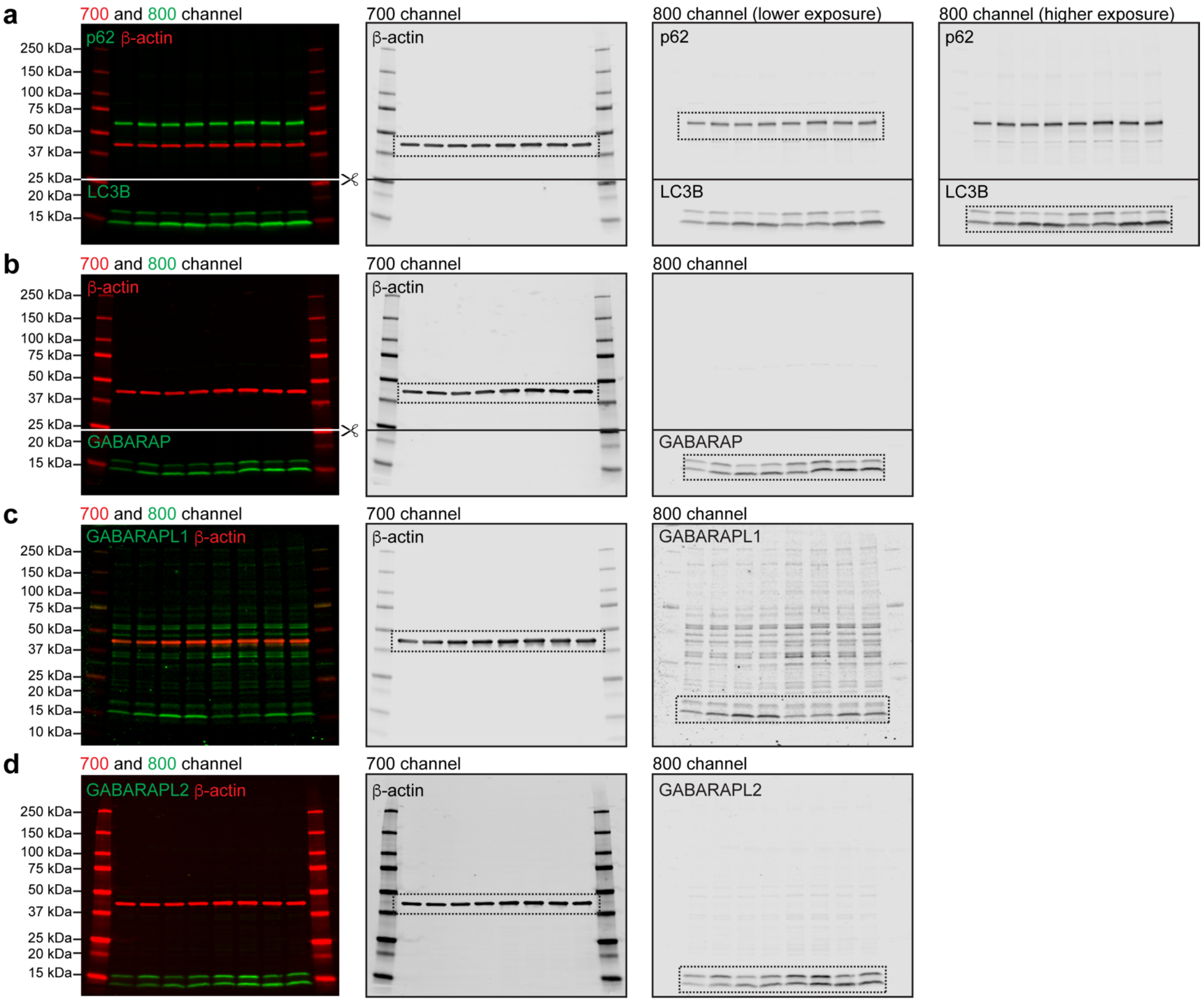

a
700 and 800 channel
p62 β-actin
LC3B
250 kDa
150 kDa
100 kDa
75 kDa
50 kDa
37 kDa
25 kDa
20 kDa
15 kDa
700 channel
β-actin
800 channel (lower exposure)
p62
LC3B
800 channel (higher exposure)
p62
LC3B
b
700 and 800 channel
β-actin
GABARAP
700 channel
β-actin
800 channel
GABARAP
c
700 and 800 channel
GABARAPL1 β-actin
10 kDa
700 channel
β-actin
800 channel
GABARAPL1
d
700 and 800 channel
GABARAPL2 β-actin
700 channel
β-actin
800 channel
GABARAPL2

Supplementary Figure 7. Uncropped images of the data presented in Figure 3b. Pseudocolored and grayscale images of the 700 and 800 channels exported from LI-COR Image Studio version 5.2.5 for p62 and LC3B (A), GABARAP (B), GABARAPL1 (C), and GABARAPL2 (D). The scissor symbol and horizontal line indicate the location where the membrane was cut, and the boxes with dotted lines indicate where the image was cropped. β-actin was used as a loading control. p62 and LC3B were assessed on the same gel. Abbreviation: kDa, kilodalton.

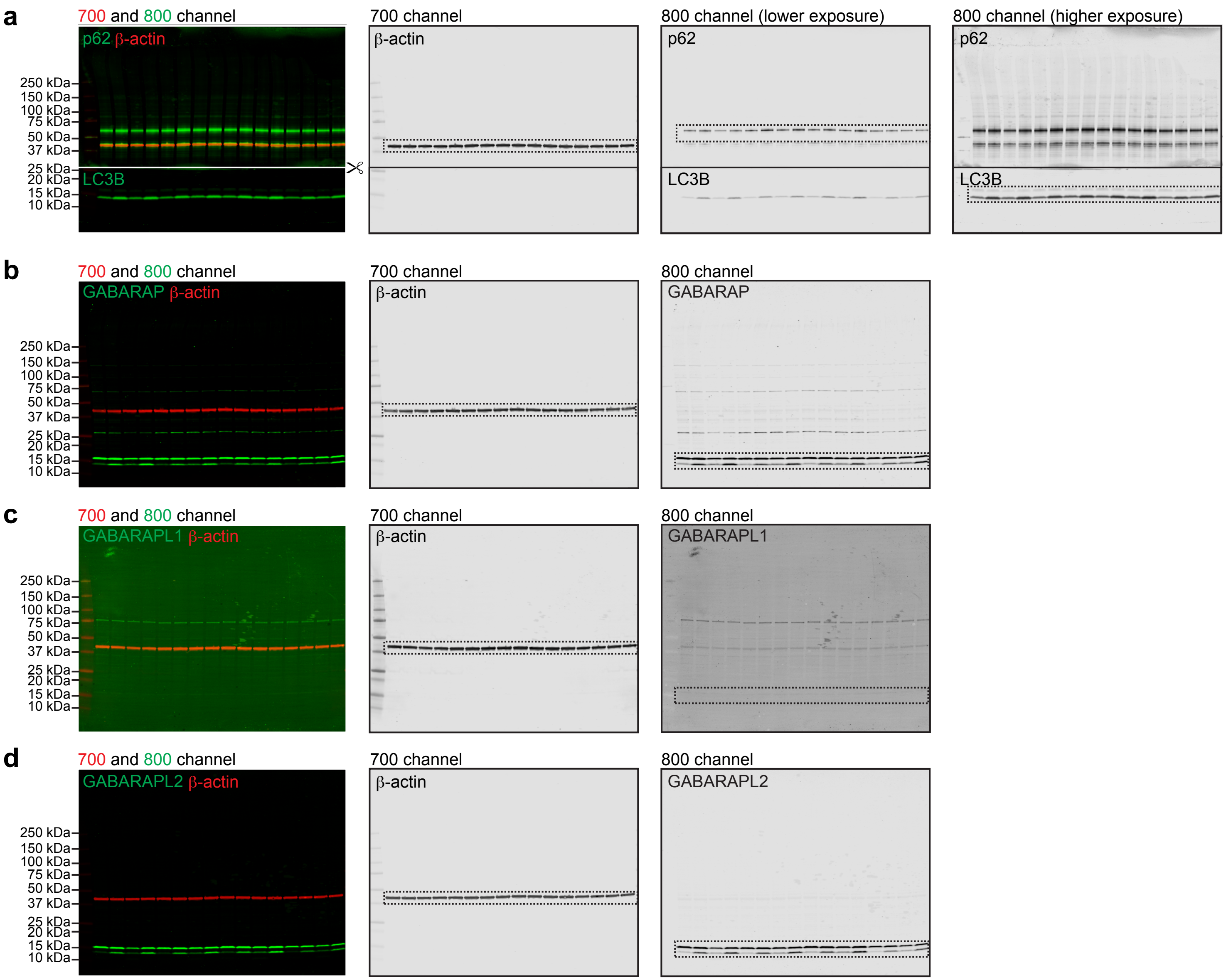
a
700 and 800 channel
p62 β-actin
LC3B
250 kDa
150 kDa
100 kDa
75 kDa
50 kDa
37 kDa
25 kDa
20 kDa
15 kDa
10 kDa
700 channel
β-actin
800 channel (lower exposure)
p62
LC3B
800 channel (higher exposure)
p62
LC3B
b
700 and 800 channel
GABARAP β-actin
700 channel
β-actin
800 channel
GABARAP
c
700 and 800 channel
GABARAPL1 β-actin
700 channel
β-actin
800 channel
GABARAPL1
d
700 and 800 channel
GABARAPL2 β-actin
700 channel
β-actin
800 channel
GABARAPL2

Supplementary Figure 8. Uncropped images of the data presented in Figure 3c. Pseudocolored and grayscale images of the 700 and 800 channels exported from LI-COR Image Studio version 5.2.5 for p62 and LC3B (A), GABARAP (B), GABARAPL1 (C), and GABARAPL2 (D). The scissor symbol and horizontal line indicate the location where the membrane was cut, and the boxes with dotted lines indicate where the image was cropped. β-actin was used as a loading control. p62 and LC3B were assessed on the same gel. Abbreviation: kDa, kilodalton.

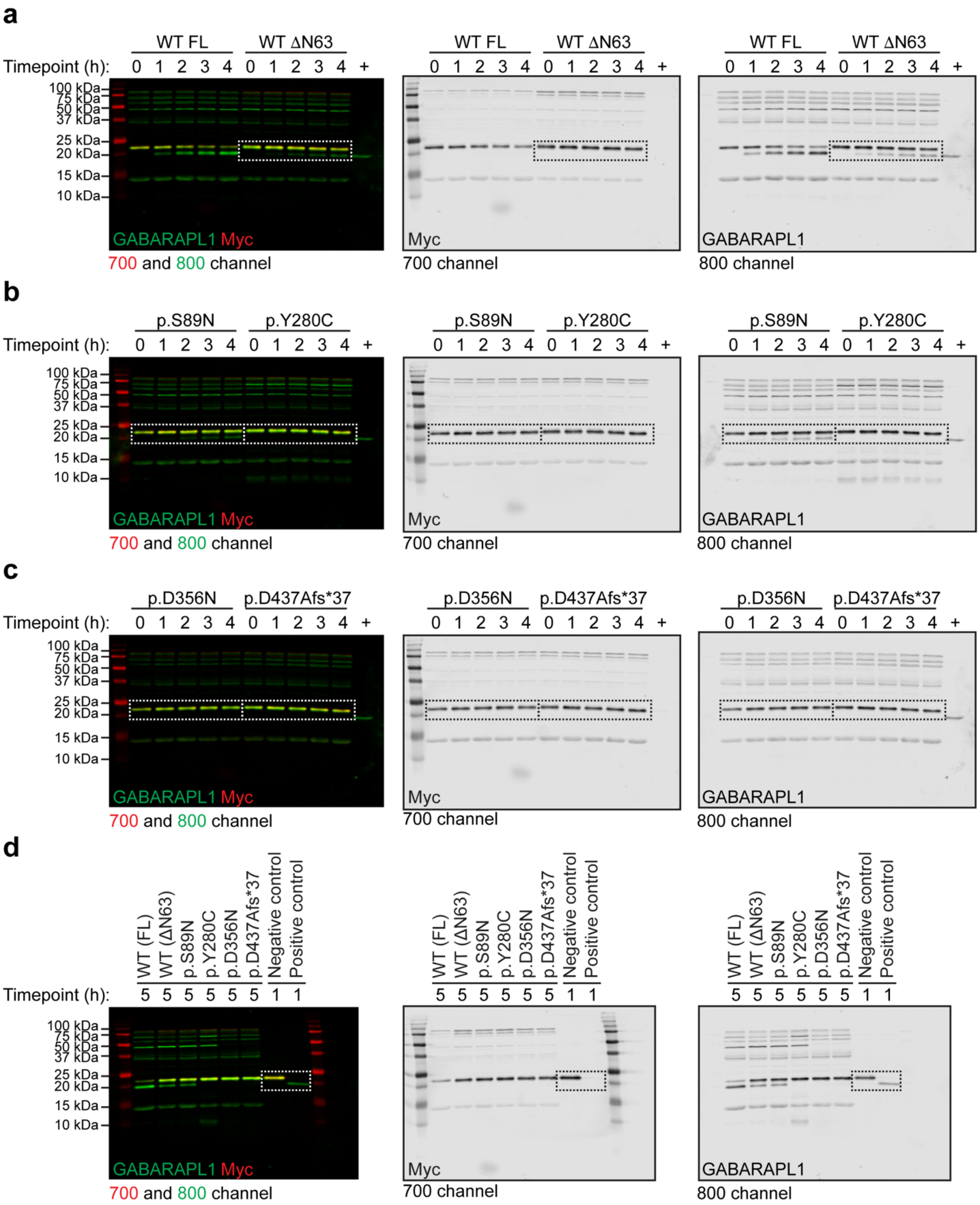

Supplementary Figure 9. Uncropped images of the data presented in Figure 4b. Pseudocolored and grayscale images of the 700 and 800 channels exported from LI-COR Image Studio version 5.2.5 to assess the *in vitro* GABARAPL1 assay products using wildtype full-length ATG4D (WT FL) and wildtype ΔN63 ATG4D (WT ΔN63) (A), p.S89N ΔN63 ATG4D

and p.Y280C ΔN63 ATG4D (B), p.D356N ΔN63 ATG4D and p.D437Afs*37 ΔN63 ATG4D (C), and each purified ATG4D for 5 h and the negative and positive controls (D). The efficient ATG4B enzyme was incubated with the G116A GABARAPL1 mutant that cannot be primed as a negative control (-) or wildtype GABARAPL1 as a positive control (+) for 1 h. The boxes with dotted lines indicate where the image was cropped. Abbreviations: FL, full-length; kDa, kilodalton; WT, wildtype.

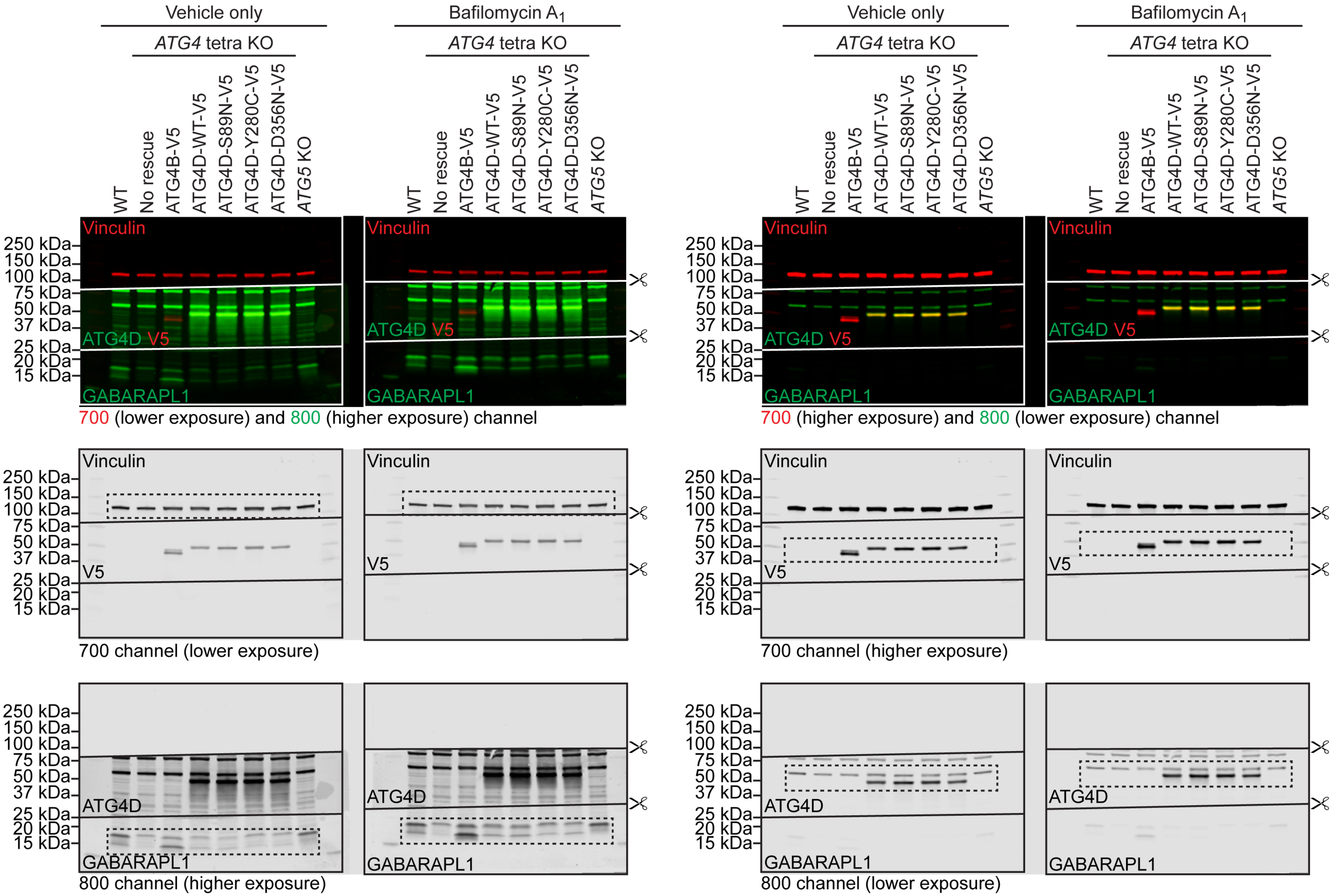

Vehicle only
Bafilomycin A1
ATG4 tetra KO
WT
No rescue
ATG4B-V5
ATG4D-WT-V5
ATG4D-S89N-V5
ATG4D-Y280C-V5
ATG4D-D356N-V5
ATG5 KO
250 kDa
150 kDa
100 kDa
75 kDa
50 kDa
37 kDa
25 kDa
20 kDa
15 kDa
Vinculin
ATG4D V5
GABARAPL1
700 (lower exposure) and 800 (higher exposure) channel
700 (higher exposure) and 800 (lower exposure) channel
V5
700 channel (lower exposure)
700 channel (higher exposure)
ATG4D
800 channel (higher exposure)
800 channel (lower exposure)

Supplementary Figure 10. Uncropped images of the data presented in Figure 4c. Pseudocolored and grayscale images of the 700 and 800 channels exported from LI-COR Image Studio version 5.2.5 to assess the priming activity of *ATG4* tetra knockout cells expressing V5-tagged wildtype (WT) ATG4D and the three ATG4D missense variants (p.Ser89Asn (p.S89N), p.Tyr280Cys (p.Y280C), and p.Asp356Asn (p.D356N)). Each column of images represents different exposures for each target (i.e., lower 700 channel exposure for vinculin, higher 700 channel exposure for V5, lower 800 channel exposure for ATG4D, and higher 800 channel exposure for GABARAPL1). The scissor symbol and horizontal line indicate the location where the membrane was cut, and the boxes with dotted lines indicate where the image was cropped. Vinculin was used as a loading control. All targets for each treatment were assessed on the same gel. Abbreviations: kDa, kilodaltons; KO, knockout; WT, wildtype.

SUPPLEMENTARY TABLES

Supplementary Table 1. Summary of bi-allelic variants identified in *ATG4D* (NM_032885.5).

Individual	Ancestry	Reported Consanguinity	Nucleotide Change	Coding Sequence Change	Amino Acid Change	Inheritance	Parent of Origin	gnomAD All	CADD phred Score
1	European	No	Chr19(GRCh37):g.10655489G>A	NM_032885.5:c.266G>A	p.(Ser89Asn)	Compound heterozygous	P	0.058%	23.1
			Chr19(GRCh37):g.10659583A>G	NM_032885.5:c.839A>G	p.(Tyr280Cys)		M	0.061%	33
2 and 3	Tatar/Russian	No	Chr19(GRCh37):g.10663628_10663646del	NM_032885.5:c.1310_1328del	p.(Asp437Alafs*37)	Compound heterozygous	P	N/A	33
			Chr19(GRCh37):g.10662741G>A	NM_032885.5:c.1066G>A	p.(Asp356Asn)		M	0.00040%	28.8

Abbreviations: CADD, Combined Annotation Dependent Depletion; gnomAD, Genome Aggregation Database; M, maternal; NA, not applicable; P, paternal.

Supplementary Table 2. Summary of missense predictions of the identified *ATG4D* variants (NM_032885.5).

Individual	Coding Sequence Change	CADD phred	REVEL	MutationTaster	M-CAP	PolyPhen-2 HumDiv	PolyPhen-2 HumVar	GERP++	phyloP 100way Vertebrate	phyloP 30way Mammalian	dbNSFP rank score
1	NM_032885.5:c.266G>A	23.1	0.12	Disease causing	Tolerated	Benign	Benign	4.4	3.242	1.176	0.501
1	NM_032885.5:c.839A>G	33	0.638	Disease causing	Damaging	Probably Damaging	Probably Damaging	5.73	8.161	1.29	0.804
2 and 3	NM_032885.5:c.1066G>A	28.8	0.896	Disease causing	Damaging	Probably Damaging	Probably Damaging	5.17	7.222	1.176	0.823

Abbreviations: CADD, Combined Annotation Dependent Depletion; dbNSFP, database for nonsynonymous SNPs' functional predictions; M-CAP, Mendelian Clinically Applicable Pathogenicity; REVEL, Rare Exome Variant Ensemble Learner; PolyPhen-2, Polymorphism Phenotyping version 2.

Supplementary Table 3. Summary of additional candidate variants identified in Individual 1.

Gene	Nucleotide Change	Coding Sequence Change	Amino Acid Change	Inheritance	Parent of Origin	gnomAD All	CADD Phred Score
PRRC2A	chr6:31599917G>A	NM_004638.3:c.3467G>A	p.(Arg1156Gln)	Compound heterozygous	M	0.09040%	25.1
	chr6:31601173G>A	NM_004638.3:c.4337G>A	p.(Arg1446His)		P	0.001442%	23.3
CADM3	chr1:159169658G>A	NM_021189.3:c.1172G>A	p.(Arg391Gln)	Compound heterozygous	M	0.001417%	29.3
	chr1:159146288G>A	NM_021189.3:c.88+4645G>A	p.?		P	0.1911%	4.5
SETX	chr9:135139307G>A	NM_015046.5:c.*319C>T	p.?	Compound heterozygous	M	0.7670%	15.6
	chr9:135205517C>T	NM_015046.5:c.1468G>A	p.(Val490Ile)		P	0.002790%	23.6
CP	chr3:148895659C>T	NM_000096.3:c.2986G>A	p.(Val996Ile)	Heterozygous	P	0.001415%	23.2
GFM2	chr5:74034420T>C	NM_032380.4:c.1127A>G	p.(His376Arg)	*de novo*	NA	0.0003985%	25.6
NRXN1	chr2:50724817G>A	NM_004801.5:c.2533C>T	p.(His845Tyr)	Heterozygous	M	0.05111%	26.1
OTOF	chr2:26706482G>A	NM_194248.2:c.1240C>T	p.(Arg414Cys)	Heterozygous	M	0.003199%	31.0

Abbreviations: CADD, Combined Annotation Dependent Depletion; gnomAD, Genome Aggregation Database; M, maternal; NA, not applicable; P, paternal.

Supplementary Table 4. Oligonucleotide primers used in this study.

**Primer Name**	**Primer Sequence (5′ to 3′)**	**Application**
ATG4D-gDNA-266-F	ACCTGTGGCAGGTCCAATAG	gDNA Sanger sequencing validation for Family 1
ATG4D-gDNA-266-R	CCTACAAGAGCGGGTCTGTC	gDNA Sanger sequencing validation for Family 1
ATG4D-gDNA-839-F	GGGGAGGAGCTTGAGAGAAC	gDNA Sanger sequencing validation for Family 1
ATG4D-gDNA-839-R	CAAACAGCAGCCCTAACACA	gDNA Sanger sequencing validation for Family 1
ATG4D-gDNA-1066-F	GCTGGGTTCTCACAGAGGAG	gDNA Sanger sequencing validation for Family 2
ATG4D-gDNA-1066-R	ACTCCTTCCTGTCTCCAGCA	gDNA Sanger sequencing validation for Family 2
ATG4D-gDNA-1310-F	ATGGACCCAAGCTGTACCGT	gDNA Sanger sequencing validation for Family 2
ATG4D-gDNA-1310-R	AGATCCCACACCCGACATGA	gDNA Sanger sequencing validation for Family 2
ATG4D-cDNA-1066-F	TTCATTGGCTACCAAGATGACT	cDNA Sanger sequencing validation for Family 2
ATG4D-cDNA-1066-R	TGTCTCCAGCATAGAAGCCC	cDNA Sanger sequencing validation for Family 2
ATG4D-cDNA-1310-F	TACCAAGATGACTTCCTGCTGT	cDNA Sanger sequencing validation for Family 2
ATG4D-cDNA-1310-R	CTGCCAGAGCTCAAGATCCC	cDNA Sanger sequencing validation for Family 2
Q5-c.266G>A-F	ACCAGCTTTAaCAAGATCTCCAG	Site-directed mutagenesis
Q5-c.266G>A-R	CCGGCTTTTAACCACCCA	Site-directed mutagenesis
Q5-c.839A>G-F	TGCACAGTGTgCAAGGCGGAT	Site-directed mutagenesis
Q5-c.839A>G-R	GTCCTGAGAAACGTACACCAC	Site-directed mutagenesis
Q5-c.1066G>A-F	GCTGTACCTGaACCCTCACTA	Site-directed mutagenesis
Q5- c.1066G>A -R	AGGAAGTCATCTTGGTAGC	Site-directed mutagenesis
Q5-c.1310_1328del-F-v1	CTGCTCCCAGCTCGCCCA	Site-directed mutagenesis (for 19-bp deletion)
Q5-c.1310_1328del-R-v1	CCTGAGCATGGCCCTCGG	Site-directed mutagenesis (for 19-bp deletion)
Q5-c.1310_1328del-F-v2	ggatgaGAAATTCGAAGCTTGGCTG	Site-directed mutagenesis (for 13-bp insertion)
Q5-c.1310_1328del-R-v2	cctccctTTATAAAAACACAAAGTCCTCAG	Site-directed mutagenesis (for 13-bp insertion)
ATG4D-RT-PCR-F	AGCCTGGAACAACGTCAAGTA	Exon 2 RT-PCR analysis
ATG4D-RT-PCR-R	CGGCCCATGTCCAGTCTCTG	Exon 2 RT-PCR analysis

Abbreviations: cDNA, complementary DNA; F, forward; gDNA, genomic DNA; R, reverse; RT-PCR, reverse transcription polymerase chain reaction.

Supplementary Table 5. TaqMan gene expression assays used in this study.

Gene	Assay ID	Targeted RefSeq Transcripts	Targeted Exon Boundary
ATG4D	Hs00262792_m1[1]	NM_032885.5<br>NM_001281504.1	6-7
ATG4D	Hs01048123_g1[2]	NM_032885.5<br>NM_001281504.1	7-8
HPRT1	Hs02800695_m1	NM_000194.2	2-3
POLR2A	Hs00172187_m1	NM_000937.4	1-2

[1]This assay is denoted as "Assay 1" in Figure S2.
[2]This assay is denoted as "Assay 2" in Figure S2.

Supplementary Table 6. Antibodies used in this study.

Antibody	Catalog No.	Company	Dilution
anti-ATG4A	7613	Cell Signaling Technology	1:1000
anti-ATG4B	13507	Cell Signaling Technology	1:1000
anti-ATG4C	ab183516	Abcam	1:1000
anti-ATG4D	ABC22	MilliporeSigma	1:1000
anti-ATG5	12994	Cell Signaling Technology	1:1000
anti-β-actin	ab6276	Abcam	1:10,000 for Figure S4<br>1:50,000 for all other figures
anti-c-myc (clone 9E10)	M4439	MilliporeSigma	1:2500
anti-LC3B	L7543	MilliporeSigma	1:1000
anti-GABARAP	13733	Cell Signaling Technology	1:1000 for Figure S6
anti-GABARAP	18723-1-AP	Proteintech	1:500 for all other figures
anti-GABARAPL1	11010-1-AP	Proteintech	1:1000 for *in vitro* assay<br>1:500 for all other figures
anti-GABARAPL1	ab229558	Abcam	1:500 for Figure S4
anti-GABARAPL2	ab122607	Abcam	1:500
anti-p62	P0067	MilliporeSigma	1:1000
anti-V5	R960-25	Invitrogen	1:2500
anti-Vinculin	V9131	MilliporeSigma	1:2000

Supplementary Table 7. gRNA target sequences used to generate the *ATG4D*-deficient HeLa cell line.

gRNA	gRNA Target Sequence
ATG4D CRISPR Guide RNA 1	ACCGTACTTGACGTTGTTCC
ATG4D CRISPR Guide RNA 5	GCTTGGGTCCATCTTGGCAA
ATG4D CRISPR Guide RNA 6	CTGCACCTCGCCCCGCAAGA

SUPPLEMENTARY NOTES

Clinical case: Individual 1

Individual 1 (Family 1: II-2) is the second child of healthy non-consanguineous parents of mixed European ancestry and first presented for evaluation at 3 years 4 months of age due to an abnormal gait, poor coordination, and staring episodes (Figure 1A and Table 1). He is the second child of healthy non-consanguineous parents. Individual 1 has an unaffected sister with a history of early language delay and concerns for pervasive developmental disorder; these symptoms, however, resolved and development progressed appropriately for her age, and she is currently a sophomore with straight A grades. The family history was otherwise unremarkable. Pregnancy was complicated by hyperemesis gravidarum and placental hematoma requiring bedrest. He was born full term via vaginal delivery and weighed 3500 g (44th centile). His early development was considered within the normal limits, and he began independently walking at 13 months. At 2 years, generalized low tone, frequent tripping and falling, and frequent staring episodes were observed. At 3 years 2 months, his mother described recurrent events characterized by ataxia, dysarthria, confusion, and behavioral changes that lasted approximately 20 minutes. During his first neurological evaluation at this time, he had mild generalized low tone that was slightly worse distally, decreased deep tendon reflexes, distal muscle weakness, a wide-based and uncoordinated gait, and reduced verbal interaction (60-75% intelligible). At 3 years 6 months, he had a seizure, described as generalized atonia with loss of consciousness (absence seizure). EEG revealed multifocal abnormalities with high risk for generalized and focal seizures and he was started on antiepileptic drugs (AED). Additional evaluations included an ophthalmological examination at 3 years 4 months that reported hyperopia, astigmatism, and amblyopia of his left eye, for which glasses were prescribed. At 4 years, increased fatigue and

somnolence were noted. At 4 years 6 months, he continued to have occasional seizures despite treatment with AED. Saccadic intrusions and central nystagmus were noted at 5 years of age. At 5 years 3 months of age, physical examination revealed slightly slow speech with dysarthria, hypotonia, intention tremor, and decreased deep tendon reflexes. His gait was wide-based, unsteady, and clumsy. Occupational therapy found neurodevelopmental deficits interfering with fine motor tasks and visual spatial difficulties. A summary of clinical findings is listed in Table 1.

Brain magnetic resonance imaging (MRI) at 3 years revealed mild cerebellar atrophy with disproportionate involvement of the superior cerebellar hemispheres and vermis; a repeat brain MRI at 5 years 3 months was ordered due to increased fatigue and progression of dyscoordination and results were stable (Figure 1B). Prolonged EEG at 5 years 3 months was normal with no focal, paroxysmal, or epileptiform abnormalities identified. A sleep study with multiple sleep latency test was consistent with hypersomnolence. At 5 years 3 months, an electromyography (EMG) and nerve conduction velocity (NCV) study revealed normal nerve conduction velocity and a lower-than-expected sural sensory nerve amplitude, and QSWEAT testing revealed a low QSWEAT response, which are consistent with a mild sensory neuropathy. CSF neurotransmitters were within normal limits. Metabolic labs were largely unremarkable. A neuropsychological evaluation at 9.5 years revealed mild cognitive impairment, ADHD (previously diagnosed at 7 years), and behavior consistent with oppositional defiant disorder.

Clinical case: Individual 2

Individual 2 (Family 2: II-3) is the third child of healthy non-consanguineous Russian parents (Figure 1A and Table 1). She has four siblings including a healthy older sister, a healthy

older brother, an affected younger sister, and an apparently healthy younger sister (Figure 1C). She was born at 37 weeks of gestation via elective Cesarean section with a birthweight of 3500 grams (91st percentile) following an uneventful pregnancy and a normal perinatal period. At 9 months, she developed episodes of abnormal hand movements characterized by short (5-10 sec) hand churning and staring episodes several times per day. She had early gross motor developmental delay, crawling at 12 months and independently walking at 20 months. At around 2 years of age, she presented with an abnormal shuffling gait with frequent tripping and falling. The hand movements spontaneously resolved by 2 years of age, and the abnormal gait has also spontaneously resolved by 4 years 8 months. On examination at 2 years 1 month, her tone was within normal limits, but at the lower end of the spectrum. She had a shuffling gait and she tripped while walking down a straight hall. Deep tendon reflexes were reduced in the patella but present (1+). She had speech delay with only 3 poorly articulated words. At 2 years 10 months, she was making steady developmental gains, but remained globally delayed. She could run a little but tripped easily. On examination at 4 years 8 months, Individual 2 had a height of 107.6 cm (69th percentile) and a weight of 16 kg (29th percentile). She had a neurological examination within the normal limits for her age and her gross motor skills are considered appropriate for her age. She did not have nystagmus, dysarthria, or coordination difficulties and her gait had normalized. Her deep tendon reflexes were normal (2+). She is currently struggling with pronunciation for which she receives speech language pathology support. She also has a mild learning disability as she struggles with number recognition and memorization.

Additional studies were performed as part of her medical evaluation for her abnormal hand movements and gross motor delay. A head ultrasound at 1 year of age showed increased extra-axial fluid at the vertex. A follow-up brain MRI at 1 year 3 months showed no remarkable

findings (Figure 1B). An EEG at 1 year 8 months was normal with no epileptiform activity identified. Metabolic and endocrine investigations showed no abnormalities. A summary of clinical findings is listed in Table 1.

Clinical case: Individual 3

Individual 3 (Family 2: II-4), the younger sister of Individual 2, was born at 32 weeks and 5 days of gestation via Cesarean section due to vaginal bleeding, preterm labor, and fetal distress (Figure 1A and C and Table 1). Her APGAR scores were 9 and 9 at one and five minutes, respectively. Her birthweight was 2263 g (86th percentile). She was admitted to the NICU for 4 weeks for transient respiratory distress, jaundice, and ongoing care. Early development showed that she had mild gross motor and speech developmental delay, but she progressively improved with her current motor and speech development considered age appropriate at age 3 years 5 months. She is currently struggling with pronunciation but there is no evidence of dysarthria. On examination at 3 years 5 months, her height and weight are within the normal limits for her age (height of 96 cm (42nd percentile) and a weight of 15 kg (58th percentile)). Her physical and neurological examination are within the normal limits for her age.

SUPPLEMENTARY DISCUSSION

Additional candidate gene variants were detected in Individual 1 (Supplementary Table 3). While some of these genes have been associated with cerebellar ataxia (*CP*) or other disorders with overlapping clinical features (*OTOF*, *GFM2*, *NRXN1*), these conditions have autosomal recessive inheritance and only one likely pathogenic allele was identified in Individual 1. Further analysis of the genome data for a second allele in *trans* did not yield additional variants for these candidate genes (Supplementary Table 3). Compound heterozygous variants were also identified in *CADM3*, encoding a brain-specific adhesion molecule, and *SETX*, a gene associated with autosomal recessive spinocerebellar ataxia with axonal neuropathy 2 (SCAN2, MIM 606002) and autosomal dominant amyotrophic lateral sclerosis (MIM 602433). However, one variant for each candidate gene was located in a deep intronic or 3′ untranslated region and with higher allele frequencies in gnomAD. Furthermore, elevated serum alpha-fetoprotein is characteristic of SCAN2[1], but Individual 1's alpha-fetoprotein levels were normal (1.3 ng/ml, normal range: 0.6 - 6.6 ng/ml). Rare, likely pathogenic compound heterozygous variants were also identified in *PRRC2A*, which encodes for the *N*-methyladenosine (m^6A) RNA modification reader that controls oligodendrocyte progenitor cell proliferation and fate determination[2]. While the gene has not been associated with any human disease, a conditional brain-specific murine knockout of *Prrc2a* was found to lead to hypomyelination, locomotive and cognitive defects, and decreased lifespan by affecting oligodendroglial specification[2]. In Individual 1, hypomyelination was not observed on MRI and nerve conduction velocity tests showed normal conduction velocities for age, suggesting normal myelination. These candidate genes were de-prioritized based on the predicted pathogenicity and population frequency data of each variant, together with the

published biological and functional data of the candidate genes and inheritance patterns of known disorders and the clinical data of Individual 1.

SUPPLEMENTARY METHODS

Immunoblot analysis to confirm total basal GABARAPL1 protein levels

Cell culture and Bafilomycin A_1 treatment of primary fibroblasts was performed as described in the main text. Cell pellets were lysed using RIPA Lysis Buffer (sc-24948, Santa Cruz Biotechnology) supplemented with Complete Mini Protease Inhibitor Cocktail (11836153001, Roche). The cysteine protease inhibitor *N*-Ethylmaleimide (E3876, Sigma-Aldrich), which has been shown to inhibit ATG4 activity and stabilize lipidated GABARAP and GABARAPL1[3], was added to a final concentration of 20 mM. The Pierce BCA Protein Assay Kit (23225, Thermo Scientific) was used for total protein quantification, and 20 μg of protein was loaded on a 4-12% gradient Bolt Bis-Tris gel (Invitrogen) for separation. Gel was then transferred to a PVDF membrane (Bio-Rad Laboratories) and blocked with 2% milk solution before incubation overnight at 4 °C with primary antibodies. Primary antibodies were diluted with Odyssey® Blocking Buffer in PBS (LI-COR Biosciences). Membranes incubated with primary antibody were washed with 1× PBS-T (0.1% Tween 20) and incubated with the appropriate secondary antibody (goat anti-mouse IgG-horseradish peroxidase (HRP) and goat anti-rabbit IgG-HRP) (Santa Cruz Biotechnology) for 1 hour. The SuperSignal™ West Femto Maximum Sensitivity Substrate (34096, Thermo Scientific) and ChemiDoc MP Imaging System (Bio-Rad Laboratories) were used to detect and visualize protein bands. Densitometry was performed using Image Lab Software (Bio-Rad Laboratories) to measure relative protein of interest present by normalizing to loading control (β-actin).

SUPPLEMENTARY REFERENCES

1 Watanabe, M. *et al.* Familial spinocerebellar ataxia with cerebellar atrophy, peripheral neuropathy, and elevated level of serum creatine kinase, gamma-globulin, and alpha-fetoprotein. *Ann Neurol* **44**, 265-269, doi:10.1002/ana.410440220 (1998).

2 Wu, R. *et al.* A novel m(6)A reader Prrc2a controls oligodendroglial specification and myelination. *Cell Res* **29**, 23-41, doi:10.1038/s41422-018-0113-8 (2019).

3 Agrotis, A., Pengo, N., Burden, J. J. & Ketteler, R. Redundancy of human ATG4 protease isoforms in autophagy and LC3/GABARAP processing revealed in cells. *Autophagy* **15**, 976-997, doi:10.1080/15548627.2019.1569925 (2019).
