# Supplementary material for: Bi-allelic ATG4D variants are associated with a neurodevelopmental disorder characterized by speech and motor impairment
Source: NPJ Genom Med. 2023 Feb 10;8:4. doi: 10.1038/s41525-022-00343-8 (PMC9918471; doi:10.1038/s41525-022-00343-8)
Supplement: Supplementary file 2 — Reporting Summary Checklist [file 41525_2022_343_MOESM2_ESM.pdf]

nature portfolio

Corresponding author(s): Malicdan

Last updated by author(s): Feb 9, 2022

Reporting Summary

Nature Portfolio wishes to improve the reproducibility of the work that we publish. This form provides structure for consistency and transparency in reporting. For further information on Nature Portfolio policies, see our Editorial Policies and the Editorial Policy Checklist.

Statistics

For all statistical analyses, confirm that the following items are present in the figure legend, table legend, main text, or Methods section.

n/a	Confirmed	
☐	☒	The exact sample size (n) for each experimental group/condition, given as a discrete number and unit of measurement
☐	☒	A statement on whether measurements were taken from distinct samples or whether the same sample was measured repeatedly
☐	☒	The statistical test(s) used AND whether they are one- or two-sided<br>*Only common tests should be described solely by name; describe more complex techniques in the Methods section.*
☒	☐	A description of all covariates tested
☐	☒	A description of any assumptions or corrections, such as tests of normality and adjustment for multiple comparisons
☐	☒	A full description of the statistical parameters including central tendency (e.g. means) or other basic estimates (e.g. regression coefficient) AND variation (e.g. standard deviation) or associated estimates of uncertainty (e.g. confidence intervals)
☐	☒	For null hypothesis testing, the test statistic (e.g. F, t, r) with confidence intervals, effect sizes, degrees of freedom and P value noted<br>*Give P values as exact values whenever suitable.*
☒	☐	For Bayesian analysis, information on the choice of priors and Markov chain Monte Carlo settings
☒	☐	For hierarchical and complex designs, identification of the appropriate level for tests and full reporting of outcomes
☒	☐	Estimates of effect sizes (e.g. Cohen's d, Pearson's r), indicating how they were calculated

Our web collection on statistics for biologists contains articles on many of the points above.

Software and code

Policy information about availability of computer code

Data collection	N/A
Data analysis	N/A

For manuscripts utilizing custom algorithms or software that are central to the research but not yet described in published literature, software must be made available to editors and reviewers. We strongly encourage code deposition in a community repository (e.g. GitHub). See the Nature Portfolio guidelines for submitting code & software for further information.

Data

Policy information about availability of data

All manuscripts must include a data availability statement. This statement should provide the following information, where applicable:

- Accession codes, unique identifiers, or web links for publicly available datasets
- A description of any restrictions on data availability
- For clinical datasets or third party data, please ensure that the statement adheres to our policy

Data that support the findings of this study are available from the corresponding author upon reasonable request. ClinVar accession codes will be available before publication.

Field-specific reporting

Please select the one below that is the best fit for your research. If you are not sure, read the appropriate sections before making your selection.

☒ Life sciences ☐ Behavioural & social sciences ☐ Ecological, evolutionary & environmental sciences

For a reference copy of the document with all sections, see nature.com/documents/nr-reporting-summary-flat.pdf

Life sciences study design

All studies must disclose on these points even when the disclosure is negative.

Sample size	No sample-size calculation was performed as this study involved patients with an ultra-rare disorder with the aim of identifying pathogenic variants in a candidate gene underlying their disorder. Once the candidate gene was identified, efforts to increase sample size were made through GeneMatcher, an online platform designed to enable connections between clinicians and researchers who share in interest in the same gene (https://genematcher.org).
Data exclusions	No data were excluded from the analyses.
Replication	Experiments were repeated or performed on different biological replicates (e.g., patient fibroblasts and/or lymphoblastoid cell lines with bi-allelic variants in ATG4D and an ATG4D-deficient cell line and appropriate controls) to verify the reproducibility of the experimental findings. All attempts at replication were successful.
Randomization	Randomization was not relevant to the study as participants were not allocated into experimental groups.
Blinding	Blinding was not relevant to the study since participants were not allocated into experimental groups.

Reporting for specific materials, systems and methods

We require information from authors about some types of materials, experimental systems and methods used in many studies. Here, indicate whether each material, system or method listed is relevant to your study. If you are not sure if a list item applies to your research, read the appropriate section before selecting a response.

Materials & experimental systems

n/a	Involved in the study	
☐	☒	Antibodies
☐	☒	Eukaryotic cell lines
☒	☐	Palaeontology and archaeology
☒	☐	Animals and other organisms
☐	☒	Human research participants
☒	☐	Clinical data
☒	☐	Dual use research of concern

Methods

n/a	Involved in the study	
☒	☐	ChIP-seq
☒	☐	Flow cytometry
☐	☒	MRI-based neuroimaging

Antibodies

Antibodies used	The antibodies used in the study include anti-ATG4D (ABC22, MilliporeSigma), anti-beta-actin (ab6276, Abcam), anti-c-Myc (clone 9E10, M4439, MilliporeSigma), anti-LC3B (L7543, MilliporeSigma), anti-GABARAP (18723-1-AP, Proteintech), anti-GABARAPL1 (11010-1-AP, Proteintech), anti-GABARAPL1 (ab229558, Abcam), anti-GABARAPL2 (ab122607, Abcam), anti-p62 (P0067, MilliporeSigma), anti-V5 (R960-25, Invitrogen), and anti-vinculin (V9131, MilliporeSigma). Additional details are noted in Supplementary Table 6.
Validation	All antibodies used in the study demonstrated the detection of a band of the expected molecular weight of their human protein target by immunoblot. Additional validation of each primary antibody is noted below.<br><br>anti-ATG4D (ABC22, MilliporeSigma) is an antibody against the N-terminus of ATG4D and a KLH-conjugated linear peptide corresponding to the N-terminus of human ATG4D was used as the immunogen. We have confirmed that this antibody specifically detects ATG4D by immunoblot through overexpression studies (examples in Supplementary Figures 2, 5, and 6). This antibody has also been knockout validated by us (see Supplementary Figure 5 and 6) and others (Kauffman et al. 2018).<br><br>anti-ATG4A, anti-ATG4B, anti-ATG4C, and anti-ATG5 has been knockout validated by the Lazarou Lab (Nguyen et al. 2021). We have confirmed this validation (see Supplementary Figure 6).<br><br>anti-beta-actin (ab6276, Abcam) has been knockout validated by Abcam.<br><br>All the following antibodies have been validated by cellular treatment with the autophagy inducer Torin 1 and the autophagy

inhibitor Bafilomycin A1 (i.e., the lipidated forms of the targets, including LC3B, GABARAP, GABARAPL1, and GABARAPL2 accumulate upon simultaneous autophagy induction and autophagy inhibition and p62 accumulates upon autophagy inhibition and decreases upon autophagy induction) by us (see Figure 3 and Supplementary Figures 3, 5, and 6), the company (noted below), and others (Nguyen et al. 2016; Chen et al. 2018; Agrotis et al. 2019 noted below).

anti-LC3B (L7543, Sigma-Aldrich) has been validated by cellular treatment by Sigma-Aldrich and Agrotis et al. (2019).
anti-GABARAP (18723-1-AP, Proteintech) has been knockout validated by Chen et al. (2018).
anti-GABARAPL1 (11010-1-AP, Proteintech) has been validated by cellular treatment by Agrotis et al. 2019.
anti-GABARAPL2 (ab122607, Abcam) has been knockout validated by Abcam and Nguyen et al. (2016) and validated by cellular treatment by Agrotis et al. (2019).
anti-p62 (P0067, Sigma-Aldrich) has been validated by cellular treatment by Agrotis et al. (2019).

anti-c-Myc (clone 9E10, M4439, MilliporeSigma) recognizes Myc-tagged proteins (see Figure 4).

anti-V5 (R960-25, MilliporeSigma) recognizes V5-tagges proteins (see Figure 4 and Supplementary Figure 6).

References

Agrotis A et al. Redundancy of human ATG4 protease isoforms in autophagy and LC3/GABARAP processing revealed in cells. Autophagy 15, 976-997 (2019).

Chen Q et al. ATL3 is a tubular ER-phagy receptor for GABARAP-mediated selective autophagy. Curr Biol 29, 846-855 (2019).

Kauffman KJ et al. Delipidation of mammalian Atg8-family proteins by each of the four ATG4 proteases. Autophagy 14, 992-1010 (2018).

Nguyen TN et al. Atg8 family LC3/GABARAP proteins are crucial for autophagosome-lysosome fusion but not autophagosome formation during PINK1/Parkin mitophagy and starvation. J Cell Biol 215, 857-874 (2016).

Nguyen TN et al. ATG4 family proteins drive phagophore growth independently of the LC3/GABARAP lipidation system. Mol Cell 81, 2013-2030 (2021).

Eukaryotic cell lines

Policy information about cell lines

Cell line source(s)	Primary dermal fibroblasts were established from forearm skin biopsies. Lymphoblastoid cell lines were established from peripheral blood and EBV transformation. Unaffected control primary fibroblasts and lymphoblastoid cell lines were obtained from Coriell Institute for Medical Research or American Type Culture Collection. HeLa (CCL-2) was obtained from American Type Culture Collection.
Authentication	Sanger sequencing of ATG4D and cellular morphology were used to authenticate the primary dermal fibroblasts or lymphoblastoid cell lines obtained from patients. All other cell lines were obtained directly from Coriell Institute for Medical Research or American Type Culture Collection and were not further authenticated.
Mycoplasma contamination	All cell lines tested negative for Mycoplasma contamination.
Commonly misidentified lines (See ICLAC register)	N/A

Human research participants

Policy information about studies involving human research participants

Population characteristics	The human research participants in the study involve a small cohort of individuals with an ultra-rare undiagnosed disease. Population-level statistics were not applied since the manuscript represents the study of an ultra-rare disorder.
Recruitment	Individual 1 was recruited through the NIH Undiagnosed Diseases Program, and Individuals 2 and 3 were identified and recruited through GeneMatcher, an online platform designed to enable connections between clinicians and researchers who share in interest in the same gene (https://genematcher.org).
Ethics oversight	The study was approved by the National Human Genome Research Institute Institutional Review Board.

Note that full information on the approval of the study protocol must also be provided in the manuscript.

Magnetic resonance imaging

Experimental design

Design type	The brain MRI images were performed at a resting state, as a part of clinical evaluation.
Design specifications	This is not applicable, as the patients were not assigned to experimental blocks

March 2021

Behavioral performance measures	This is not applicable, as the patients were not assigned to experimental blocks

Acquisition

Imaging type(s)	Structural
Field strength	3
Sequence & imaging parameters	Images were acquired in T1 and T2 sequences.
Area of acquisition	Whole brain scan

Diffusion MRI ☐ Used ☒ Not used

Preprocessing

Preprocessing software	Data were viewed through a standard DICOM viewer.
Normalization	Normalization was not done
Normalization template	This was not done
Noise and artifact removal	This was not done
Volume censoring	This was not done

Statistical modeling & inference

Model type and settings	This was not done
Effect(s) tested	This was not done

Specify type of analysis: ☐ Whole brain ☐ ROI-based ☐ Both

Statistic type for inference (See Eklund et al. 2016)	Not applicable
Correction	Not applicable

Models & analysis

n/a	Involved in the study
☒	☐ Functional and/or effective connectivity
☒	☐ Graph analysis
☒	☐ Multivariate modeling or predictive analysis
